# Retrieving functional pathways of biomolecules from single-particle snapshots

**DOI:** 10.1038/s41467-020-18403-x

**Published:** 2020-09-18

**Authors:** Ali Dashti, Ghoncheh Mashayekhi, Mrinal Shekhar, Danya Ben Hail, Salah Salah, Peter Schwander, Amedee des Georges, Abhishek Singharoy, Joachim Frank, Abbas Ourmazd

**Affiliations:** 1 Department of Physics, University of Wisconsin Milwaukee, 3135 N. Maryland Ave, Milwaukee, WI 53211 USA; 2 Beckman Institute for Advanced Science and Technology, University of Illinois at Urbana-Champaign 405 N. Mathews Ave., Urbana, IL 61801 USA; 3 School of Molecular Sciences, Center for Applied Structural Discovery, Arizona State University, Tempe, AZ 85287 USA; 4 Structural Biology Initiative, CUNY Advanced Science Research Center, City University of New York, New York, NY 10031 USA; 5 Department of Chemistry & Biochemistry, City College of New York, New York, NY 10031 USA; 6 Ph.D. Programs in Physics, Chemistry & Biochemistry, The Graduate Center of the City University of New York, New York, NY 10016 USA; 7 Department of Biochemistry and Molecular Biophysics, Columbia University, 2-221 Black Building, 650 West 168th Street, New York, NY 10032 USA; 8 Department of Biological Sciences, Columbia University, 600 Fairchild Center, New York, NY 10027 USA

**Keywords:** Computational biophysics, Computational models

## Abstract

A primary reason for the intense interest in structural biology is the fact that knowledge of structure can elucidate macromolecular functions in living organisms. Sustained effort has resulted in an impressive arsenal of tools for determining the static structures. But under physiological conditions, macromolecules undergo continuous conformational changes, a subset of which are functionally important. Techniques for capturing the continuous conformational changes underlying function are essential for further progress. Here, we present chemically-detailed conformational movies of biological function, extracted data-analytically from experimental single-particle cryo-electron microscopy (cryo-EM) snapshots of ryanodine receptor type 1 (RyR1), a calcium-activated calcium channel engaged in the binding of ligands. The functional motions differ substantially from those inferred from static structures in the nature of conformationally active structural domains, the sequence and extent of conformational motions, and the way allosteric signals are transduced within and between domains. Our approach highlights the importance of combining experiment, advanced data analysis, and molecular simulations.

## Abstract (web summary)

There is a great interest in retrieving functional pathways from cryo-EM single-particle data. Here, the authors present an approach that combines cryo-EM with advanced data-analytical methods and molecular dynamics simulations to reveal the functional pathways traversed on experimentally derived energy landscapes using the ryanodine receptor type 1 as an example.

## Introduction

In principle, a macromolecule with *N* atoms has (3*N-6)* degrees of conformational freedom. Barring exceptions such as intrinsically disordered proteins, biological function involves coordinated changes in structural blocks, e.g., an alpha helix, or an entire molecular domain. Conformational motions can thus be described in terms of a small number of so-called conformational coordinates, each describing the concerted motions of a large number of atoms. The choice of conformational coordinates is not unique; the bending of a person’s arm, for example, can be described in terms of the angle subtended by the elbow, or the hand-to-shoulder distance. No choice of conformational coordinates is inherently linear, but mutually orthogonal coordinates are the most convenient. All choices will reveal the same movements, but not necessarily at the same local rate of change. The number and nature of the degrees of freedom exercised during unperturbed function, and the conformational coordinates relevant to function must be extracted from the data.

In equilibrium, each conformational state of a macromolecule is occupied with a statistical weight, ${\mathrm{e}}^{ - \left( {E_i/k_BT} \right)}$, where *E*_*i*_ is the free energy of the conformational state *i*, *k*_*B*_ the Boltzmann constant, and *T* the temperature. Given appropriate sampling of the conformational space, single-particle snapshots of a sufficiently large number of macromolecules will include all thermally accessible conformational states. The energy of each observed conformational state can be determined from the number of times it has been sighted, through the so-called inverse-Boltzmann relation between the statistical weight and the energy of the state^1–3^ (see also “Methods”).

It has long been recognized that energy landscapes offer a powerful framework for studying conformational changes in macromolecules (see refs. ^4–6^), with function unfolding along heavily populated minimum free-energy paths^7–11^. Based primarily on molecular simulations, this realization has yielded important insights into biological function, ranging from protein folding to small-molecule binding and motor action^12,13^.

The majority of experimentally determined energy landscapes have involved one conformational coordinate^14,15^, have been compromised by low spatial resolution^16,17^, and/or limited by the inability to account for the multiple energy landscapes associated with the vast variety of biological functions involving ligands and cofactors^17^.

The combination of recent resolution improvements in cryo-EM with new data-analytical and molecular simulation techniques now offers an unprecedented opportunity to identify functional paths on multiple experimentally determined energy landscapes, and compile all-atoms movies of complex biological functions, including those involving ligands and cofactors.

At present, cryo-EM is widely used to infer functional information from static structures obtained by powerful maximum-likelihood classification methods^18,19^, which sort single-particle snapshots into a user-defined number of discrete conformational clusters. These methods incorporate continuous conformations as admixtures of orientationally independent, rigid domain structures^20^. In general, the positions and sequence of these structures in the macromolecular work cycle are unknown, and it is not always easy to assess the relevance of these structures to function. Under such circumstances, the functional inference is based on interpolations between static structures, if only conceptually. In the absence of additional information, arranging such discrete structures along a functionally relevant pathway is difficult. Since the number of ways in which two discrete structures can be transformed into each other is essentially unlimited, reliable functional inference by discrete clustering is not straightforward.

The primary goals of this paper are as follows: (i) demonstrate that energy landscapes associated with complex biological function can be extracted from experimental data, and corroborated by molecular simulations, (ii) elucidate the conformational paths associated with complex biological function in all-atoms detail, including possible routes for transitions between different landscapes, (iii) establish that motions associated with functional paths on energy landscapes can be significantly different from those inferred by discrete clustering methods, (iv) outline the new biological insights gained by studying the continuous conformational changes associated with function, and (v) render the algorithms used in this paper widely accessible.

We use cryo-EM single-particle snapshots of ryanodine receptor type 1 (RyR1) to exemplify the discovery process facilitated, and the new insights revealed by our approach. RyRs are calcium-activated calcium channels critical to excitation/contraction coupling in heart and skeletal muscle. Malfunctions in RyR1 and RyR2 can lead to calcium leaks deleterious to heart and skeletal muscle function^21^. Insights into RyR channel function and regulation are therefore critical in understanding the role of disease-causing mutations and identifying pharmacological leads^21^.

The architecture of the ryanodine receptor can be divided into three major regions: the channel pore, responsible for calcium efflux from the sarcoplasmic reticulum; an activation core, which binds activating ligands and is responsible for channel activation; and a large cytoplasmic shell serving as a platform for the binding of many regulatory proteins^22^. Ca^2+^, ATP, and caffeine are well-characterized activators of RyR, synergistically activating the channel by inducing a rotation of its activation core and pore opening. Still, the allosteric mechanism by which rotation of the activation domain renders pore opening possible remains unknown. We therefore set to reanalyze with our manifold-based geometric machine-learning approach^17,23–26^ the ligand-free snapshots and those with all three ligands (Ca^2+^, ATP, and caffeine) previously examined by clustering techniques^22,27^.

Our results allow us to contrast the ligand-binding pathways revealed by our approach^17,23–26^ with results obtained from the same dataset by interpolating between discrete RyR1 structures obtained by clustering techniques (see refs. ^22,27–30^). This comparison reveals major differences in the delineation of functionally active structural domains, the nature, sequence, and extent of motions associated with RyR1 function, and the propagation of allosteric signal to functionally important remote sites.

## Results

### Energy landscapes

The 791,956 cryo-EM snapshots of RyR1 molecules analyzed in this study comprised about the same number of molecules in equilibrium with a thermal bath with and without ligands (Ca^2+^, ATP, and caffeine) prior to cryo-freezing^22^ (see “Methods”). These snapshots were first aligned to a common 3D reference by standard iterative procedures implemented in RELION^19^. Based on these alignment parameters, snapshots were grouped into 1117 uniformly spaced orientational bins for manifold-based analysis (see “Methods”). The RyR1-EGTA structure EMD-8391 was filtered to 40-Å resolution and used for orientation recovery for both data sets.

Assuming the association dynamics of the ligands are faster than the functionally relevant conformational motions of the protein^31^, pooling data from the two experiments allows both RyR1 species (with and without ligands, henceforth ± ligand) to be described in terms of the same set of conformational coordinates. Manifold-based analysis^17,26^ of the pooled data revealed four conformational coordinates above the noise plateau, each describing a particular set of continuous conformational changes. A further detailed analysis was restricted to only the two strongest conformational coordinates (CC1 and CC2 for short) because the lower-power coordinates were too weak to yield meaningful results (see “Methods”). Broadly speaking, conformational changes along CC1 involve the cytoplasmic shell, the activation core, and the pore; those along CC2 involve only the cytoplasmic shell (See Supplementary Note 1 and Supplementary Fig. 9).

**Fig. 1:**
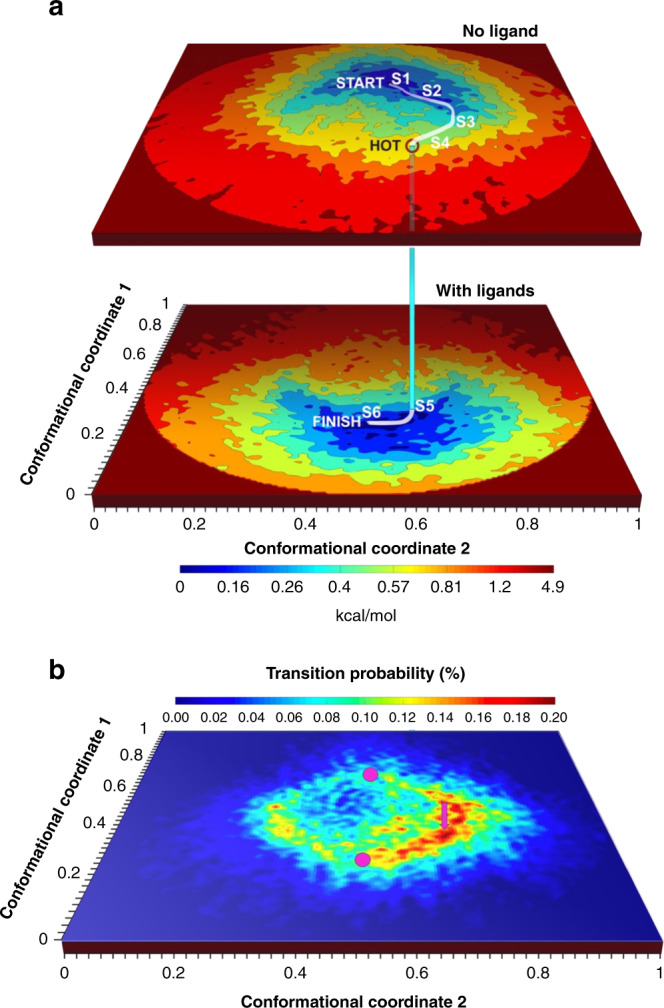
Energy landscapes with and without ligands, and transition probability map. **a** Energy landscapes without ligands (upper surface), and with ligands (Ca^2+^, ATP, and caffeine) (lower surface). The landscapes are described in terms of the most important two of a common set of orthogonal conformational coordinates. The curved path represents a high-probability route to the binding of ligands. This path starts at the minimum-energy conformation of RyR1 without ligands (“START”), follows the conduit of lowest energy to a point with a high probability of transition to the with-ligands energy landscape (“HOT”), and terminates at the minimum-energy conformation with ligands (“FINISH”). Six representative conformational states along the path, in white, are selected for validation with molecular dynamics simulations (S1–S6). **b** Probability map for transitions from the energy landscape without ligands to the energy landscape with ligands. The axes are the same as in Fig. 1a, with magenta discs indicating the positions of the minima of the energy landscapes. The magenta arrow indicates the position of the transition point.

The resulting ±ligand energy landscapes (Fig. 1a) reveal the heavily populated, functionally important conformational conduits^7–11^ relevant to ligand association and binding. Molecular simulations are then employed to elucidate the association pathways and reversible binding kinetics (see below).

### Molecular movies of ligand binding in RyR1

Based on a master equation approach^32,33^, we estimate the probability of a transition between iso-conformational points on the two landscapes shown in Fig. 1a to be the product of the density of occupied states on the upper landscape and the density of unoccupied states on the lower landscape (see “Methods”). This estimate is expected to be reasonable, as long as ligand dynamics are fast compared with the rate of conformational changes in RyR1. Of course, association pathways derived from this approach offer an incomplete description of the binding pathway. Capturing the reversible binding process requires a proper description of the ligand-dissociation dynamics, which involves coupled protein motions, and is often rate-determining^34^.

Subject to the above limitations, the interlandscape transition probability displays specific “hotspot” regions (Fig. 1b). The most probable routes to ligand association then start from the region of lowest energy on the –ligand landscape (“START” in Fig. 1a), reaching one of the hotspot transition zones (“HOT”) with a probability of ~2%. (To be clear, this value refers to the probability of finding the hotspot occupied on the upper landscape, not the interlandscape transition probability shown in Fig. 1b.)

**Fig. 2:**
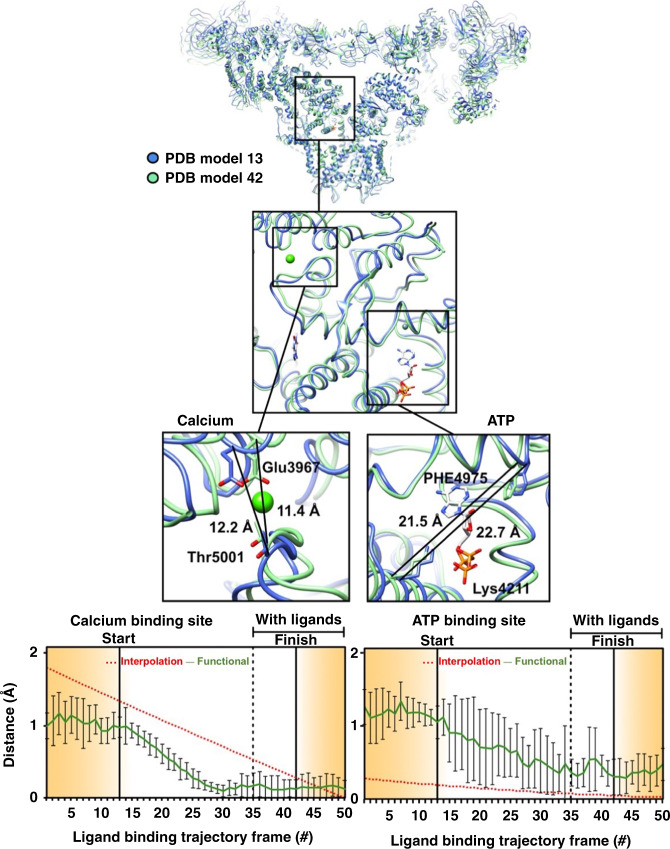
Conformational changes at calcium and ATP-binding sites along the functional path. Ryanodine receptor 1 (RyR1) conformational changes at calcium and ATP-binding sites along the functional trajectory of Fig. 1a, augmented with excursions from the minimum-energy points along CC1. These excursions are in the increasing CC1 direction on the no-ligand landscape, and in the decreasing CC1 direction on the with-ligand landscape. Top: The general region and the specific sites examined in detail. Distance variations between two amino acid backbones on the opposite side of the ATP and calcium are shown. The amino acids used for measurement are represented as sticks, the rest of the molecule in blue ribbon for the model corresponding to “START” in Fig. 1a, and in green ribbon for model corresponding to “FINISH”. Bottom: Distance variations between carbon-alpha backbone atoms of two opposing residues at each of the calcium and ATP binding sites for 50 structures along the functional and interpolated trajectories. For the latter, 50 structures were extracted from a morph between a model of RyR1 without ligands (PDB: 5TB4) and a model of RyR1 with calcium, ATP, and caffeine (PDB: 5T9V). To analyze the functional route, six published models (PDB: 5TB4, 5T9R, 5TAP, 5T9V, 5TAL, 5TAQ) were each fitted and refined into the maps along the trajectory. Error bars represent the full scatter (not standard deviation) of the results obtained with different starting models.

**Fig. 3:**
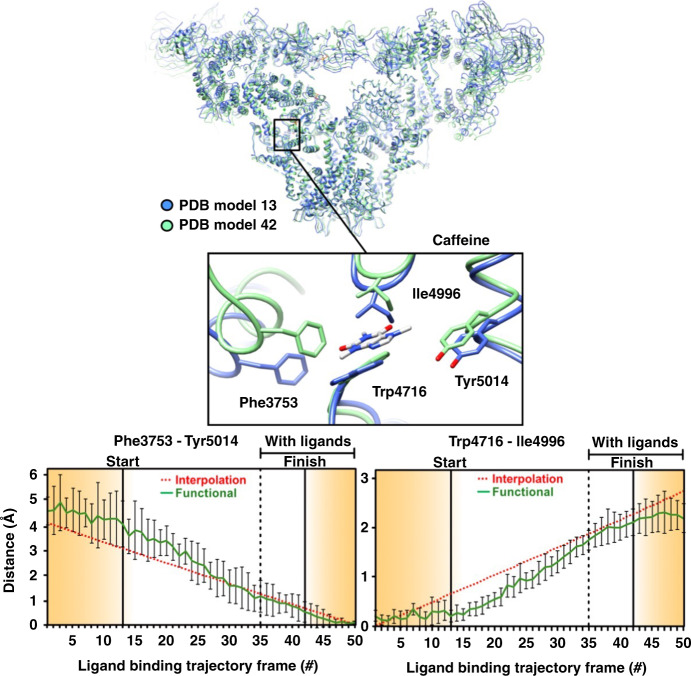
Conformational changes at the caffeine binding site along the functional path. Ryanodine receptor 1 (RyR1) conformational changes at the caffeine binding site along the functional trajectory of Fig. 1a, augmented with excursions from the minimum-energy points along CC1. These excursions are in the increasing CC1 direction on the no-ligand landscape, and in the decreasing CC1 direction on the with-ligand landscape. Top: The general region and the specific site (inset) are examined in detail. The molecule is shown in blue ribbon for the model corresponding to “START”, and in green ribbon for model corresponding to “FINISH” in Fig. 1a. Bottom: Distance variations between two pairs of amino acid backbones on the opposite side of caffeine. The amino acids used for measurement are represented as sticks. Error bars represent the full scatter (not standard deviation) of the results obtained with six different starting models.

**Fig. 4:**
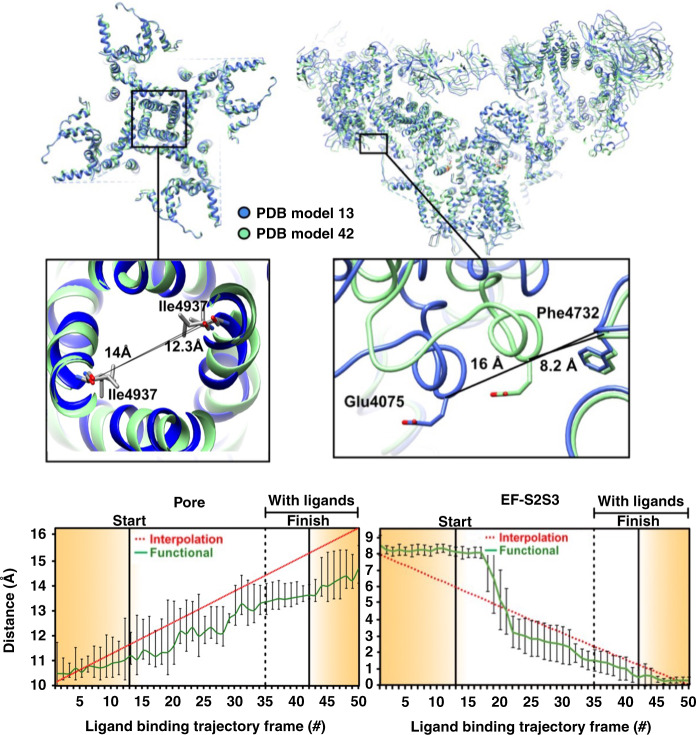
Conformational changes in the pore and EF-hand domains along the augmented functional path. The general region and the specific sites (insets) are examined in detail. Distance variations between two amino acid backbones: in the pore measured at Ile4937 (left); and between EF hand (EF) at Glu4075 and S2S3 domain at Phe4732 (right). The amino acids used for measurement are represented as sticks, the rest of the molecule in blue ribbon for the model corresponding to “START” in Fig. 1a, and in green ribbon for model corresponding to “FINISH”. Error bars represent the full scatter (not standard deviation) of the results obtained with six different starting models.

**Fig. 5:**
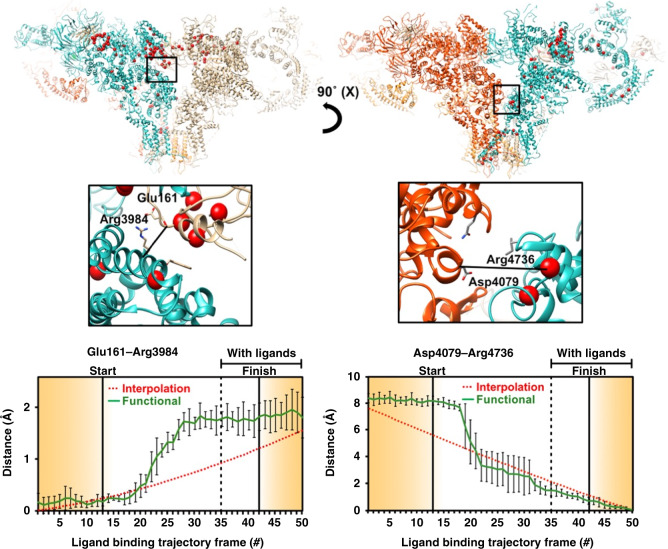
Conformational changes at interprotomer contact sites along the augmented functional path. The monomers are shown in different colors. Red markers highlight known point-mutations involved in malignant hyperthermia-1 and central core disease of muscle. Distance variations are between two amino acid backbones at contact sites between the monomers. The amino acids used for measurement are shown as sticks. Distance variations between carbon-alpha backbone atoms of two opposing residues along the augmented functional path. Error bars represent the full scatter (not standard deviation) of the results obtained with six different starting models.

At the hotspot, the trajectory crosses over to the +ligand landscape with ~0.24% of the probability of association with a ligand, terminating in the region of lowest energy on the +ligand landscape (“FINISH”). Three-dimensional (3D) movies compiled along heavily populated conduits from the START point on the −ligand landscape to FINISH point on the +ligand landscape (the white curve in Fig. 1a) reveal the conformational motions relevant to ligand association, in some regions with near-atomic resolution (Supplementary Movies 1–6, see “Methods” for more detail). Conformational changes at the binding sites of the ligands Ca^2+^, ATP, and caffeine as ligand binding proceeds are represented in Figs. 2–5 (The 50-frame movies of conformational changes along the route connecting the two minima include excursions from these minima to the “north” and “south” on the upper and lower landscapes, respectively, in order to facilitate accurate distance measurements at key binding sites (see below)).

We note, however, that the binding probability, while correlated with the association probability, can demonstrate very different trends. We therefore use MD simulations to establish the validity of the ligand-association landscape derived directly from cryo-EM^35^.

### Molecular dynamics simulations

The movies of ligand association were further refined in all-atoms detail by molecular dynamics (MD), multi-conformation continuum electrostatics (MCCE), and free-energy simulations (see “Methods”). Building upon the visualization of the association conduits (irreversible pathways of ligand entry into the binding pockets) directly from experimental data, MD reveals the mechanism of reversible ligand binding (capturing the quasi-equilibrium ensemble of structures along the ligand entry and exit pathway). This free-energy analysis was augmented by MCCE, which monitors the change in ligand-binding affinity along the conduit. Thus, starting with cryo-EM data, the thermodynamics of a reversible binding process is captured in MD simulations, elucidating ligand association, dissociation, and coupled transitions of the protein (see “Methods”).

MD simulations were performed at six successive points along the functional path of Fig. 1a connecting the START and FINISH points. These simulations indicate that the high-energy cost of Ca^2+^ binding renders binding unlikely between the conformational states 1 and 2 (S1 and S2) (Supplementary Note 3 and Supplementary Figs. 1–3). In qualitative agreement with the experimentally estimated association probabilities, the binding interactions become favorable from state 3 (“S3”) onward, becoming most probable at the FINISH conformation (Fig. 1a). In the absence of an associating Ca^2+^, the energy of a transition from S1 → S6 increases, corroborating the experimentally determined trend in Fig. 1a (Supplementary Fig. 1). The rapid decrease in conformational energy and binding affinity between states 2 and 3 implies minimal protein conformational changes during the Ca^2+^ association. As illustrated further in Supplementary Fig. 4, the binding affinity of Ca^2+^ to each one of the cryo-EM models calculated by MCCE reveals that the energy cost of switching between the +ligand and –ligand landscapes is initially high, but rapidly decreases as the interlandscape transition region is approached.

### Functional paths vs. interpolation between discrete clusters

The curved nature of the route to binding (the white line connecting START to FINISH in Fig. 1a) shows that conformational motions relevant to binding involve an elaborate and changing admixture of conformational coordinates, which cannot be determined from the START and FINISH structures alone. This underscores the difficulty of deducing functional information from discrete structures.

**Fig. 6:**
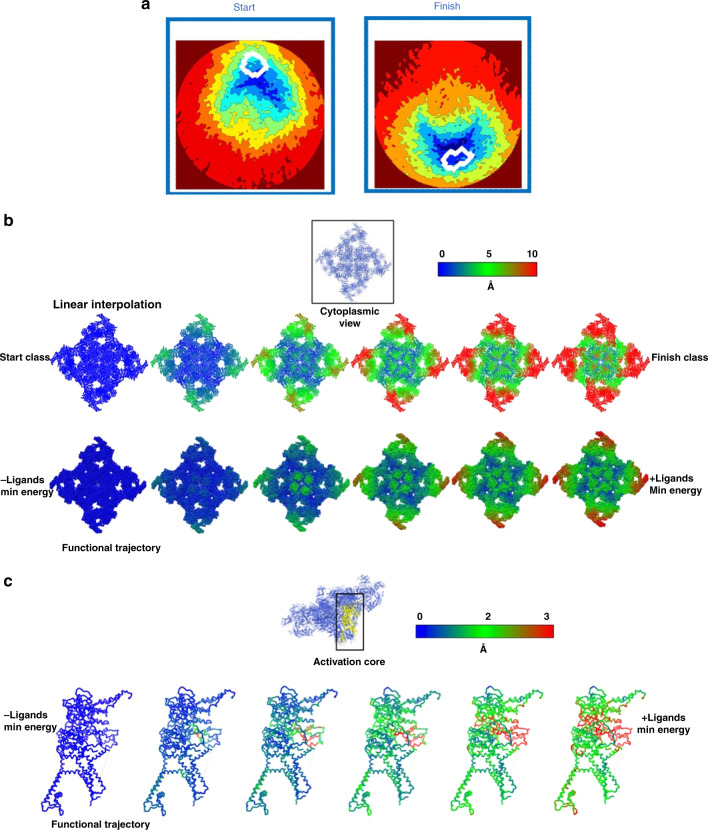
Functional path vs. interpolation between discrete clusters. **a** Distribution on the energy landscape of Fig. 1a of snapshots assigned by maximum likelihood to two discrete conformational clusters. Each white curve encloses a region of high snapshot density. These two classes were previously identified as representing the extremes of the conformational range, and used to infer function by discrete cluster analysis^22^. **b** Overall conformational changes along the functional path vs. linear interpolation between the two maximum-likelihood classes previously used for functional inference^22^ (see panel **a**). The ryanodine receptor 1 (RyR1) atomic model is shown in sphere representation, with color indicating the displacement of each atom from its “START” position. **c** Conformational changes in the activation core domain (residues: 3614-5037) along the functional path of Fig. 1a. The RyR1 backbone structure is shown as sticks, with the color of each atom indicating its atomic displacement (see color bar). The largest atomic displacements along the functional trajectory are confined to a narrow allosteric conduit connecting the ligand-binding sites to the EF hand.

The experimentally determined energy landscapes make it possible to compile molecular movies along functional paths, and compare the results with the changes inferred by maximum-likelihood clustering^36^ of the same snapshots^22,27^. The RyR1 data analyzed here were previously clustered with RELION 3D classification into 16 discrete conformational classes, 2 of which were labeled as “junk”^22^. For each class, the positions and distribution of the snapshots on the energy landscapes are shown in Fig. 6a and Supplementary Fig. 6. It is difficult to discern a systematic relationship between the positions of the different discrete classes on the energy landscapes. Class 2 (no ligands) and class 3 (with ligands) were taken in the previous cluster-based analysis^22,27^ to represent the functionally relevant extremes of the conformational range. Functional information was then inferred by interpolating between the 3D structures obtained from these two classes. Below we compare the conformational changes deduced from such interpolation with the changes associated with the functional trajectory on the energy landscapes (Fig. 1a).

Before doing so, however, we note two important points. First, the extent to which maximum-likelihood clustering is based on functionally relevant conformational coordinates is unknown. The position of a discrete cluster on the relevant energy landscape (Fig. 6a) thus represents a projection from an unknown space onto space spanned by the two most important conformational coordinates relevant to ligand binding. As a result, the minimum-energy conformations (START and FINISH) observed on the energy landscapes may differ from the conformations observed with discrete clustering. Second, interpolation (“morphing”) between two or more static structures along a putative functional path is widely acknowledged as invalid, but nonetheless often used, because discrete classification methods provide no information about the functional path traversed between the different discrete structures. Sometimes elastic network models or steered or targeted MD are employed to derive functional pathways. While better than linear morphs, such approaches are also known to manifest serious free-energy artifacts.

We now turn to the conformationally active structural domains and their motions. Figure 6 and Supplementary Movies 3 and 4 compare the displacements revealed along the functional path (“functional analysis” for short) with those inferred from interpolating between the two discrete classes, viz. ligand-free closed, and ligand-bound open conformations, as described in a previous study^22^. These two discrete classes lie close to the energy minima on the ±ligand energy landscapes, i.e., the START and FINISH points of the functional path. As such, the comparison offers the most optimistic assessment of functional inference from discrete clusters.

There are major differences between the results obtained from landscape-based functional analysis and those inferred from interpolation between the discrete clusters. These differences include the structural domains involved in motion, as well as the sequence and extent of displacements (Fig. 6b, c and Supplementary Movies 3–6). For example, in contrast to the results from cluster analysis^22,27^, functional analysis shows that: (a) the N-terminal domains (NTD) lead the sequence of motions; (b) a significant part of the macromolecule remains rigidly static during function; and (c) the motions in the activation core and shell are coupled (Fig. 6b and Supplementary Movies 3–6).

## Discussion

The conformational changes derived from functional analysis are significantly different from those inferred by interpolating between the discrete structures, even though the clusters used are close to the termini of the functional path. For example, while the importance of NTD for RyR1 function has long been recognized^37–39^, conformational changes specific to the NTD have not been described in previous high-resolution cryo-EM studies, or have specific elements of the shell been shown to be more rigid than others^22,29,40^.

Notably, the energetically uphill motions of opening the Ca^2+^-binding pocket in the –ligand state cannot be captured by brute-force targeted MD simulations, when only the START and FINISH conformations are provided. Information from at least six structures along the functional route on the energy landscapes was needed to identify a thermally accessible pathway by MD-based piecewise free-energy simulations (see “Methods”). This clearly demonstrates that discrete cluster analysis, even augmented with MD, is unable to trace a meaningful functional pathway.

We now discuss the implications of our results for the allosteric mechanisms responsible for channel pore opening upon binding of activating ligands. This discussion is facilitated by distance measurements at important sites, which quantify the consequences of functional motions.

First, our results elucidate longstanding questions regarding “population shift” vs. “induced fit” models of allosteric ligand binding. Broadly speaking, “population shift”^41^ requires a conformational change before, “induced fit”^42^ a conformational change after ligand binding. The higher-energy conformations are occupied thermally via “population shift”. The exact apportionment of the conformational changes before and after association depends, of course, on the point at which the transition to the +ligand landscapes takes place. Although transitions can occur over a relatively broad region, the highest association probabilities are concentrated at a few “hotspots” (Fig. 1b). The positions of the ±ligand energy minima relative to the broad region of significant transition probability indicate that most ligand-binding events in RyR1 involve a greater element of “population shift” than “induced fit”, as suggested by Nussinov et al.^43^.

Second, Figs. 2–5 and Supplementary Figs. 10–13 reveal the conformational changes at the binding sites of Ca^2+^, ATP, and caffeine, as ligand binding proceeds (see Supplementary Note 2). It is known that ligand binding stabilizes the activation domain in a conformation suitable for pore opening^22^. Our results show this activated state can also be present in the ligand-free state, albeit with low or no conductance. Consistent with the independently measured low probability of channel opening in the absence of calcium and ATP^44^, the energy landscape shows only a small fraction of RyR1 molecules assume the activated conformation in the ligand-free state. Further pore opening requires the binding of a ligand followed by an “induced fit” to the minimum-energy conformation of the ligand-bound receptor. The relatively broad +ligand energy minimum (Fig. 1a) is also consistent with other measurements: Brownian motions of the shell^27–29^ give rise to conformational dispersion along CC2; and the spikes in conductance, interpreted as channels flickering between the open and closed states^44^, cause dispersion along CC1.

Third, despite extensive work (see ref. ^22^), it has proved difficult to clarify how the conformational changes associated with ligand binding in the activation domain lead to gating and pore opening. Our distance measurements reveal potentially important atomic motions as the functional trajectory is traversed, most strikingly along a previously unobserved allosteric conduit connecting the ligand-binding sites in the Csol domain to the EF hand (Fig. 4). Frame-by-frame measurements indicate the displacements begin at the calcium-binding site, and propagate along a narrow “vein” to the EF-hand (Fig. 6c and Supplementary Movies 5, 6). The movement of the EF hand described above is in line with earlier observations^22,29^. Our analysis uncovers a previously unobserved allosteric conduit between the ligand-binding sites and the EF hand, identifying the mechanical motions underlying signal transduction (Supplementary Movie 5). In contrast, the displacements inferred from discrete cluster analysis are distributed uniformly over large regions, with no special feature indicating targeted signal transduction (Supplementary Movie 6). The functional analysis shows the narrow band of displacements first appears on the –ligand landscape, i.e., before ligand binding. This further supports “population-shift” in the first part of the ligand-binding process, whereby a ligand stabilizes fleeting conformational fluctuations present before ligand binding.

The observed coupling of the EF-hand movement to ligand binding highlights the potential role of the EF hand in gating, or in its regulation. This movement leads to an interaction between the EF-hand and the S2S3 domain of the pore pseudo-voltage sensor. The small movement of the S2S3 domain associated with the EF-hand pair interaction suggests that this subtle movement of S2S3 may be relevant to gating, and should be further investigated. As noted earlier, the NTD is among the first domains affected by the transition between ligand-free and ligand-bound states (Fig. 6b and Supplementary Movies 3, 4), indicating a potentially important role in gating for NTDs. Indeed, the NTDs give rise to important interprotomer interactions, which are lost during channel dilation and subsequent pore opening, and a number of disease-causing mutations are located at these interfaces^38,45,46^. It is thus important to understand whether NTDs and other inter-domain contacts involved in the gating mechanism are destabilized by the binding of ligands prior to pore opening, or they are sufficiently weak to be broken by Brownian motions during pore opening, a mechanism known as the “zipper hypothesis”^47^. To clarify this question, we investigated the distance between interprotomer contacts as the functional path is traversed. The analysis was limited to backbone-to-backbone distances, as the resolution of our present study is limited by the number of available snapshots to ~4.5 Å in the core of the channel, precluding reliable measurement of side-chain positions (see “Methods”).

Two interprotomer contacts display significantly nonlinear behavior, suggesting that they are modulated by ligand binding and may have a possible role in gating (Figs. 4 and 5). The first such contact is formed between the EF-hand and the S2S3 domain of the neighboring protomer, as outlined above. We observe a stepwise motion bringing these two domains into close proximity well before the transition to the ligand-bound state and pore opening (Fig. 4). The EF-hand pair movement is, therefore, correlated with channel activation, rather than pore opening. This observation further supports the observation made earlier of an allosteric conduit between the calcium-binding site and the EF hand, and points to an important functional role for the EF-hand pair, which has thus far remained elusive. It has been shown that the deletion of the EF hand does not affect channel activation by calcium^48^. The EF-hand pair may instead either have a critical role in the channel regulation by other proteins^49^, or in channel inactivation at high calcium concentrations^50^. The fact that the EF-hand pair is allosterically linked to the activation domain means that a ligand binding to the EF-hand pair and influencing its conformation could have a strong influence on calcium affinity to the activation domain, and therefore on channel activation by calcium. This allosteric relationship between the EF-hand pair and activation domain may explain the sometimes contradicting results obtained on the role of the EF-hand pair in channel activation by calcium^48–50^.

The second significant contact is situated between the NTD β8–β9 loop and the activation domain. These two domains move apart by ~1.5 Å in a stepwise fashion as the interlandscape transition point is approached (Fig. 5). The loop, containing a number of charged residues suitable for ionic interactions, has been suggested to play an important role in gating^38,46^. This contact is thus a “gate” candidate, where strong ionic interactions would prevent pore opening before ligand binding. The contact would be broken by conformational changes in the activation domain upon ligand binding to allow pore opening. Other, weaker “zipper” interactions would then be broken and reformed at intervals by the Brownian motion of the channel, accounting for the observed “flickering” behavior of RyR1. This offers an important hypothesis for the RyR1 gating mechanism.

In a number of positions, the distance change between interprotomer contact points shows either very small motions, or a linear behavior. For instance, distance measurements between opposite residues at the pore gate (Ile4937) show an approximately linear increase from the beginning to the end of the reaction path (Fig. 4). We also note that the beginning and endpoints of the “functional” trajectory are different from those of the two extreme classes selected from the RELION classification (Fig. 4, interpolation). The distance change between Calpha of opposite protomers shows a pore dilation of ~4 Å along the least-action path, which is about 2 Å less than the dilation observed between the classes chosen from the clustering analysis and to the previous reports^22^. While this may seem surprising, it should be noted that the previous analysis focused on the transition between free and bound ligand states, and did not explore the energy landscape of each dataset independently. The path explored goes through the minimum-energy point of each landscape, which probably corresponds to a partially open state for the minimum energy of the ligand-bound state. Further exploration of the conformational landscape of the ligand-bound state by itself, or of the pore domain more specifically would probably uncover the full range of pore radii, but this is outside the scope of this study.

Finally, the IP3 receptor, with a homologous calcium activation mechanism, does not have EF-hands and an S2S3 domain, but its NTD are homologous to the RyR1 NTDs, where the activating ligand IP3 binding site is located^51,52^. The IP3 receptor could thus have a homologous mechanism, by which binding of calcium and IP3 leads to conformational changes in the activation domain and NTD of IP3R, followed by pore opening.

As noted earlier, inferring a biological function from the structure is a paramount goal of structural biology. The results presented here highlight the importance of basing functional inference on energy landscapes and conformational coordinates derived from the data. Our approach is based on three concepts of general significance. First, biological function involves a rich set of continuous conformational changes inadequately described by discrete structures of unknown relationship. Linear interpolations between the X-ray or cryo-EM structures can produce a qualitative understating of some large-scale motions, but almost always fail to reveal the changes along the minimum free-energy pathway of a conformational transition^35^. Elastic network^53^ or flexible-fitting methods represent the next logical steps, but the energetics, and functional pathways of protein-ligand binding are difficult to resolve with these methods. Second, thermal fluctuations in equilibrium lead to sightings of all states up to an energy limit set by the number of snapshots in the dataset. This makes it possible to compile the energy landscapes needed for a rigorous description of the thermodynamics of function. This data-guided approach can be further enhanced with important sampling methods, such as string simulations with swarms of trajectories^54^, Markov state models^55^, meta-dynamics *a la* meta-inferencing^56^, or adaptive biasing forces applied here. No external steering is required to induce a rare event. Third, conformational changes underlying complex biological function can be determined from experimental data by combining advanced data analytical techniques with powerful simulation approaches. We believe the power of our approach is demonstrated by the energy landscapes, the interlandscape transition maps, the new information on conformationally active structural domains, and the nature, sequence, and extent of important displacements involved in function. The approach is applicable to a wide range of systems and processes.

## Methods

### The upper limit on the energy of accessible conformational states

The difference in the Gibbs free energy Δ*G* between two states with populations *N*_*A*_ and *N*_*B*_ is given by the Boltzmann relation, viz. *N*_*B*_*/N*_*A*_ = exp (−Δ*G/k*_*B*_*T*), with *k*_*B*_ the Boltzmann constant, and *T* the absolute temperature. Using an ensemble of *N* snapshots, the state sighted only once lies at an energy Δ*G*_max_ above the lowest energy state of the ensemble. Under these conditions, *N*_*B*_ = 1 and *N*_*A*_ = *N* −1 ≈ *N*. Thus, the highest energy observed in an ensemble of *N* particles corresponds to Δ*G*_max_ = *k*_*B*_*T* log *N*. For the dataset analyzed in this paper, *N*_*−*ligand_ = 293,619 and *N*_+ligand_ = 262,022, yielding a theoretical upper limit Δ*G*_max_ *~*7 kcal/mol. This inverse-Boltzmann approach for deducing free energies performs well and is used widely (see refs. ^1–3,16^).

### Effect of coarse graining on the energy landscapes

Here we address the effect of coarse-graining on the resulting energy landscapes. From Maxwell–Boltzmann statistics of noninteracting particles in thermal equilibrium, the occupation probability of a discrete state *c* is given by:

1 $$P_c = \frac{{g_ce^{ - E_c/KT}}}{Z},{\mathrm{with}}\,{\mathrm{partition}}\,{\mathrm{sum}}\; Z = \sum {g_ce^{ - E_c/KT}} .$$

*g*_c_ is the degeneracy of the state *c*, specifically, the number of experimentally indistinguishable conformational states assigned to energy *E*_*c*_, which may nevertheless be distinguished from each other by some other means, *E* = *U, H*, or *G*, (internal, Helmholtz, or Gibbs free energy, respectively). The sum extends over all possible states.

Conformational sorting yields the number of sightings (snapshots) of each conformation. Each sighting represents a conformational state occupied in thermal equilibrium. A conformational bin contains all conformations indistinguishable by the experimental and data-analytical pipeline used (“coarse-graining”).

The experimental observable, namely the number of sightings of a conformational state *c* is given by:

2 $$n_c = N_{{\mathrm{snapshots}}}Z^{ - 1}g_ce^{ - \,\frac{{E_c}}{{KT}}}.$$

In principle, Eq. (2) provides a direct link between the number of sightings (snapshots) of a conformation, and its energy.

However, the degeneracy *g*_*c*_ induced by coarse-graining is unknown, and, in general, conformation-dependent. *g*_*c*_ can be absorbed into the exponent via an entropy term, viz.

3 $$\begin{array}{l}n_c = N_{{\mathrm{snapshots}}}Z^{ - 1}e^{ - \left( {\frac{{E_c - TS_c^{cg}}}{{KT}}} \right)} = N_{{\mathrm{snapshots}}}Z^{cg^{ - 1}}e^{ - \left( {\frac{{E_c^{cg}}}{{KT}}} \right)}.\, S_c^{cg} = k\, log\, g_c\end{array}$$

with the superscript *cg* short for coarse-graining.

Equations (2) and (3) implicitly assume that all conformations coarse-grained into the same conformational class have the same energy. This need not be the case. Without further information or assumptions, it is not possible to determine energy differences between conformational classes from Eqs. (2) or (3).

Mapping conformational landscapes without regard to the degeneracy induced by coarse-graining (as commonly practiced) is predicated on the further assumption that the variance of *E*_*c*_ dominates over that of $TS_c^{cg}$, viz.

4 $$\mathop {\sum}\limits_{c} {(\left\langle {E_c} \right\rangle - E_c)^2} \gg T\mathop {\sum}\limits_{c} {(\left\langle {S_c^{cg}} \right\rangle - S_c^{cg})^2}.$$

In this work, Eq. (3) is used subject to the assumption that Eq. (4) holds.

### Input data, preprocessing steps, and analytical pipeline

The details of the data used for the present analysis have been described elsewhere^22,27^. Here, we provide a brief outline for the reader’s convenience.

### Rabbit skeletal muscle RyR1 purification

Purified RyR1 was prepared from rabbit (*Oryctolagus cuniculus*) skeletal muscle using the following procedure, modified from^57^ Snap-frozen rabbit skeletal muscle (100 g) was blended in cold buffer containing 10 mM Tris-maleate pH-6.8, 1 mM DTT, 1 mM EDTA, 150 μM PMSF, and 1 mM benzamidine, and centrifuged for 10 min at 8000×*g*. The supernatant was centrifuged for 20 min at 40,000×*g*. Pellets were solubilized in 50 ml of buffer containing 10 mM HEPES pH = 7.5, 1% CHAPS, 1 M NaCl, 2 mM EGTA, 2 mM TCEP, and protease inhibitors cocktail (Roche). The solubilized membranes were then diluted 1:1 in the same buffer without the NaCl, and centrifuged for 30 min at 100,000×*g*. The supernatant was then passed through a 0.2-micron filter and allowed to bind overnight at 4 °C to a pre-equilibrated 5-ml GSTrap (GE Healthcare) column with bound GST-Calstabin1. The column was then washed with modified solubilization buffer (0.5% CHAPS and 0.5 M NaCl), and RyR1 was eluted with two column volumes of 10 M calstabin2 in the same buffer. The protein was eluted using calstabin2 (also known as FKBP12.6), rather than the physiological binding partner calstabin1 (FKBP12), owing to the higher affinity of the former for the RyR1 channel. The eluent was pre-cleared with 0.5 ml glutathione beads and treated with calf intestinal alkaline phosphatase (CIP, NEB, 100 U ml^−1^) for 4 h at room temperature. RyR1 was then concentrated on a 100,000 kDa cutoff centrifugation filter and run through a size-exclusion column (tosoh G4SWxl) with a solubilization buffer with the following alterations and substitutions: 5 mM EGTA, 0.25% (w/v) CHAPS, and 0.001% (w/v) DOPC (Avanti). The mono-disperse peak was then concentrated on a 100,000 kDa cutoff centrifugation filter to ~5–10 mg ml^−1^.

### Residual Ca^2+^ concentration in the no-ligands solution

The buffer (0.5 M NaCl) was potentially contaminated with 0.002% Ca^2+^, corresponding to 10 μM Ca^2+^ in solution. In total, 5 mM of EGTA was added to chelate the solution. A small concentration of Ca^2+^ ions remains in the no-ligands solution after such treatment. According to https://somapp.ucdmc.ucdavis.edu/pharmacology/bers/maxchelator/CaMgATPEGTA-TS-Plot.htm, this free Ca^2+^ concentration is 0.2 nM. Similar concentrations are reported in http://onlinelibrary.wiley.com/doi/10.1113/jphysiol.1968.sp008413/epdf.

We now investigate whether binding of residual Ca^2+^ contamination is responsible for displacing the RyR1 molecules from the no-ligand minimum-energy region to functionally significant, higher-energy regions of the no-ligands landscape. Ca^2+^ contamination in “no-ligands” solution: 2 × 10^−10^ M, Total RyR1 in “no-ligands” solution (5 mg ml^−1^, 2.3 MDa): 2 × 10^−6 ^M, number of RyR1 at no-ligands transition point (2% of total): 4 × 10^−8^ M, Assuming all Ca^2+^ is bound, the ratio [no. of Ca^2+^-contaminated RyR1 molecules]*/*[no. of RyR1 molecules at transition point] is: 2 × 10^−10^/4 × 10^−8^ = 5 × 10^−3^ = 0.5%.

The concentration of Ca^2+^ contaminants is ~0.5% of the number of RyR1 molecules in functionally important, high-energy regions of the landscape, such as the interlandscape transition points. The role of any Ca^2+^ contamination is thus negligible.

### Cryo-EM

It should be noted that in this study, we used the cryo-EM micrographs from two previous studies^22,27^, in which the RyR-EGTA and RyR-30 μM Ca^2+^-ATP-caffeine samples were prepared on holey carbon grids (C-flat CF-1.2/1.3-2C-T, Protochips Inc, NC). In all, 3 μL of each sample was applied to holey-gold grids, blotted for 3.5–4 s and vitrified by rapidly plunging into liquid ethane with a Vitrobot (FEI). Data were acquired using an FEI Tecnai F30 Polara (FEI, Eindhoven) operating at 300 kV with the automated data collection software Leginon^58^ on a K2 Summit direct electron detector camera (Gatan, Pleasanton, CA) at a nominal magnification of ×31,000, corresponding to a calibrated pixel size of 1.255 Å. For experiments using carbon grids, images were recorded in dose-fractionated mode, each image being fractionated into 20 frames. The total exposure time was 4 s, yielding a total accumulated dose of 25 electrons/Å^2^ on the specimen. The beam diameter was set at ~500 nm in order to capture two images per hole using the image shift. As normal in cryo-EM, only processes slow compared with the freezing time can be faithfully captured.

### Image processing

Dose-fractionated image-stacks collected with a Gatan K2 Summit camera were aligned using MotionCorr^59^, and the sum of aligned frames was used for further preprocessing. The particles were picked with RELION 1.3^19^ reference-based automated particle-picking procedure^60^, and their defocus values were estimated by ctffind4^61^. Particles were subjected to 3D classification using RELION 1.3 to select the good particles. This process removed particles that did not have the appearance of a receptor. In this way, 366,000 particles from the ligand-free data set and 450,000 particles from the ligand-bound data set were selected. No symmetry was imposed in the 3D classification and particle-picking stage. Each data set was then further classified in 3D into eight classes without symmetry imposed to observe the structural heterogeneity of the particles, and compare these results with the outcome of analysis by the manifold approach.

### Orientation recovery

The orientation parameters for each of the two data sets were refined separately using RELION 1.3 with the same starting reference and no symmetry imposed. The RyR-EGTA structure EMD-8391 was filtered to 40-Å resolution and used for orientation recovery of both data sets. These alignment parameters were subsequently used for the manifold-based analysis, as described in refs. ^17,62^. In brief, aligned, centered snapshots were divided into projection directions, specifically, into groups falling onto tessellations of a spherical shell subtending a semi-cone angle of two Shannon angles (defined as spatial resolution (0.4 nm)*/*particle diameter (32 nm)).

### Geometric (manifold-based) analytical pipeline

Following orientation recovery, snapshots from RyR with and without ligands were pooled and analyzed (“embedded”) together. Under these circumstances, the algorithm finds the conformational coordinates best able to describe both datasets. If the two data sets are “very different”, the algorithm yields two well-separated clusters. Each cluster can then be investigated independently in terms of its own coordinates.

Embedding the snapshots in each projection direction by Diffusion Map^63^ revealed two clusters, one corresponding to an “artifact class” of unusually low contrast (Supplementary Fig. 7a). This class was excluded, leaving 791,956 snapshots for further analysis. The manifold obtained from these remaining snapshots is shown in Supplementary Fig. 7b.

The manifold-based analytical pipeline, including the effect of noise, is described in detail in ref. ^17^. In outline, the pipeline consists of the following steps: diffusion map embedding of ±ligand snapshots in each projection direction, nonlinear Laplacian spectral analysis (NLSA)^24^ along the eigenfunctions for each projection direction, conformational coordinate propagation across projection directions, metric homogenization across projection directions, compiling the energy landscape for each of the ±ligand data sets with snapshots in all projection directions, mapping the interlandscape transition probability over the landscape, using the formalism described below, compiling 3D movies along functional trajectories on the energy landscapes. To validate the approach, we simulated a system with two degrees of freedom, generated synthetic cryo-EM snapshots with and without noise for many projection directions, and combined the information from these projection directions to obtain the final energy/occupancy landscape (Supplementary Methods and Supplementary Figs. 14–16).

### Limitations

We note that the alignment procedure leads to some signal loss in two ways:

All particles are aligned iteratively to a common reference in RELION. This common reference represents the average of all conformations present in the datasets. In such cases, particles will align primarily either to the rigid set of domains with the largest mass, to the center of mass of the complex, or to a combination of both. In the case of RyR1, both pore and cytoplasmic domains are large, and particles are aligned approximately to the center of mass of the molecule. The center of mass corresponds approximately to the core of the channel, with the extremity of the cytoplasmic shell blurred out by differences in conformation between particles. But because the cytoplasmic shell represents a large portion of the channel mass, the center of mass is also affected by changes in the conformation of the cytoplasmic shell. As a result, the pore of the channel may be somewhat misaligned. Because particle alignment is passed onto the subsequent steps of the analytical pipeline without further refinement, these caveats need to be considered in the analysis and interpretation of the output data. Particle misalignment with respect to a particular domain will also dampen the observed conformational differences in this domain. To increase the signal from subtle conformational differences within specific domains, all particles should be aligned to these domains independently prior to the geometric analytical pipeline. While recognizing the issue, we believe such an analysis is beyond the scope of the present paper.

The fact that the geometric analytical pipeline is performed on aligned, centered snapshots assigned to projection directions means that the signal from conformational variability is dampened by the width of the Shannon angle used to define the angular width of each projection direction. The signal from conformational variability of small amplitude may therefore be lost. This could explain why only two conformational reaction coordinates were consistently above the noise plateau in this analysis.

### Estimating transition probabilities

Master equations describe the dynamics of transitions between states^32^. We write a master equation as follows:

5 $$\frac{d}{{dt}}p_c\left( t \right) = \mathop {\sum }\limits_{c^{\prime} = 1}^N w_{cc^{\prime}}p_{c^{\prime}}\left( t \right) - p_c(t)\mathop {\sum }\limits_{c^{\prime} = 1}^N w_{cc^{\prime}}.$$

Here, *p*_*c*_(*t*) is the time-dependent probability of a particle being in state *c*. The first term on the right-hand side is the rate of gain in state *c* due to transitions from other states, the second term the rate of loss from state *c* to other states, and *w*_*cc’*_ are the transition rates. *N* is the total number of states.

For ligand binding, we consider each of the *N* conformational states to be either bound or unbound to a ligand. The master equation can be formulated as two coupled equations, one for the probability with ligand (+) and the other without ligand (−).

6 $${\frac{{{d}}}{{{{d}}t}}p_c^ + \left( t \right)} = \, {\mathop {\sum }\limits_{c^{\prime} = 1}^N W_{cc^{\prime}}^{ + + }p_{c^{\prime}}^ + \left( t \right) + \mathop {\sum }\limits_{c^{\prime} = 1}^N W_{cc^{\prime}}^{ - + }p_{c^{\prime}}^ - (t) - p_c^ + (t)\mathop {\sum }\limits_{c^{\prime} = 1}^N (W_{cc^{\prime}}^{ + + } + W_{cc^{\prime}}^{ + - })} \\ {\frac{{{d}}}{{{{d}}t}}p_c^ - \left( t \right)} = \, {\mathop {\sum }\limits_{c^{\prime} = 1}^N W_{cc^{\prime}}^{ - - }p_{c^{\prime}}^ - \left( t \right) + \mathop {\sum }\limits_{c^{\prime} = 1}^N W_{cc^{\prime}}^{ + - }p_{c^{\prime}}^ + (t) - p_{c}^{-} (t)\mathop {\sum }\limits_{c^{\prime} = 1}^N (W_{cc^{\prime}}^{ - - } + W_{cc^{\prime}}^{ - + })}.$$

The transition rates can be from states bound to bound (++), unbound to bound (−+), bound to unbound (+−), and unbound to unbound (−−), as denoted by their superscripts.

Consider the initial stages of the approach to equilibrium with the ligand reservoir. At times short compared with the ensemble relaxation time, the conformational spectrum is nearly the same as that prior to contact with the ligand reservoir.

7 $${p_c^ + \left( {t = 0} \right)} = \, 0 \\ {p_c^ - \left( {t = 0} \right)} = \, p_{c,eq}^{-} .$$

The equation for the bound states thus becomes initially:

8 $$\left. {\frac{{{{d}}p_c^ + }}{{{{d}}t}}} \right|_{t = 0} = \mathop {\sum}\limits_{c^{\prime} = 1}^N {W_{cc^{\prime}}^{ - + }p_{c^{\prime},eq}^ - }.$$

The transition rates $W_{cc{\prime}}^{ - + }$ are in general unknown. Here, we assume ligand binding connects iso-conformational states on the −ligand and +ligand landscapes. In this model, the interlandscape transition per se is too rapid to allow conformational adjustments, but maybe predicated on prior conformational changes, and/or initiate subsequent conformational adjustments. The expression for the gain of the bound state *c* yields:

9 $$\left. {\frac{{{{d}}p_c^ + }}{{{{d}}t}}} \right|_{t = 0} = \mathop {\sum}\limits_{c^{\prime} = 1}^N {W_{cc^{\prime}}^{ - + }} p_{c^{\prime},eq}^ - \delta _{cc^{\prime}} = W_{cc^{\prime}}^{ - + }p_{c^{\prime},eq}^ -.$$

The transition rate $W_{cc{\prime}}^{ - + }$ will be conformation dependent in general. To obtain an estimate, we use the principle of mass action kinetics for ligand binding^33^, viz.

10 $$\left. {\frac{{{{d}}p_c^ + }}{{{{d}}t}}} \right|_{t = 0} = k_cLp_c^ -.$$

*L* is the free-ligand concentration, and *k*_*c*_ is the association rate constant, which characterizes the velocity of the second-order interaction between the receptor and the ligand. Note that dissociation does not enter the equation because there are no bound receptors initially. The free-ligand concentration initially is the same for all conformations, so the conformation dependence is due to *k*_*c*_ only. We estimate the association rate constant by

11 $$k_c = Ae^{\frac{{ - E_a}}{{KT}}},$$

with activation energy $E_a = E_c^ + - E_{c0}^ +$ from the energy landscape with ligands, where $E_{c0}^ +$ is the lowest energy. This yields:

12 $$k_c = A\frac{{P_c^ + }}{{P_{c0}^ + }} \propto P_c^ +.$$

And with Eq. (10),

13 $$\left. {\frac{{{{d}}p_c^ + }}{{{{d}}t}}} \right|_{t = 0} \propto P_c^ + P_c^ -.$$

As a consequence, the maximum association and consequently transition occurs at the conformation which makes the product of the two conformational spectra maximal. The quantities $P_c^ +$ and $P_c^ -$ can be estimated from the number of snapshots in each conformational bin, as deduced from the conformational analysis of the two data sets.

14 $$P_c^ + = \frac{{n_c^ + }}{{N_c^ + }},\,P_c^ - = \frac{{n_c^ - }}{{N_c^ - }}.$$

The accuracy of this estimate and location of the (−+) $\leftarrow\rightarrow$ (++) transition hotspots is limited by the number of snapshots in the experiment, and coupling of the protein and ligand conformations in the dissociation pathway that is not accounted for in this model.

This result is in line with transition probability determination between + and – surfaces using nonequilibrium switches^64^, albeit assuming small conformational changes. The rapid decrease in conformational energy and binding affinity between states 2 and 3, observed in our MD simulations and further described in Supplementary Fig. 4, implies minimal protein conformational changes during the Ca^2+^ association, justifying the assumption.

### Molecular dynamics simulations

The conformational states S1–S6 selected for molecular dynamics study represent different points along the functional trajectory revealed by the data-analytical pipeline (Fig. 1a). The states start at the minimum-energy point [“START”] on the –ligand energy landscape, terminating at the minimum-energy point [“FINISH’] on the +ligand landscape. Each state is associated with a particular density map along the minimum-energy path. Ca^2^^+^-binding domains of the six RyR1 conformational states were solvated with TIP3P water and neutralized with 100 mM NaCl (Supplementary Movies 7 and 8).

In the molecular simulations, the Ca^2^^+^ ions were initially placed following the MCCE procedure, outlined below. Initial equilibration was performed with NAMD2^65^ in an NPT ensemble with periodic boundary conditions. The simulations were performed at 310 K using Langevin dynamics^66^ with a damping constant of 0.5 ps^−1^. The Nosé–Hoover Langevin piston method^66^ was used to maintain constant pressure at 1 atm. The cutoff used for the short-range interactions was 12 Å with the switching applied at 10 Å. The particle mesh Ewald (PME) algorithm^67^ was used to calculate the long-range electrostatic force. Bonded, non-bonded, and PME calculations were performed at 2, 2, and 4 fs intervals, respectively.

For every state S1 to S6, the Ca^2^^+^-binding activation core domain (defined as residues 3747–5035) was truncated from the rest of the protein, mainly the cytosolic shell. Thereafter, the system was minimized for 5000 steps using the conjugate-gradient algorithm, and simulated for 5 ns at 310 K, with all the heavy atoms of the protein restrained to their initial positions with force constant of *k* = 5 kcal/mol/Å^2^. Finally, all the restraints were removed, and the systems simulated for 100 ns, prior to the production runs of an additional 100 ns described above. Their respective stability was tracked by calculating the backbone root-mean-square deviation (RMSD) of the conformations sampled with respect to the starting structure. As can be seen in Supplementary Fig. 2a, the converged RMSD with respect to the starting conformation is between 2 and 3 Å, corroborating the relative stability of the Ca^2+^-binding domain simulated.

A closer look at the binding site revealed sub-1 Å changes in the binding pocket due to truncation. This observation stemmed from our frame-by-frame RMSD matrix across structures from all the six simulated states from S1 to S6 (Supplementary Fig. 2b). Put together, all the RMSD data suggest the stability of the truncated models used in the production run and subsequent free-energy simulations. As expected, states S5 and S6 being the most stable in the Ca^2+^-bound form show the least scatter on the plot.

### Ligand association and binding

Association of the Ca^2^^+^ ion to RyR1 was studied by equilibrating Ca^2^^+^ ion for 100 ns in the calcium-binding sites corresponding to the conformational states S1–S6. Ca^2^^+^ is most mobile in state S1, with the relative mobility progressively decreasing from states S1 to S6. This is manifested in the distribution of the distances between the Ca^2^^+^ ion and the binding site residues as one progresses from S1 to S6. As shown in Supplementary Fig. 4, in state S1, the Ca^2^^+^ is unstable with a broad distance distribution, while in the state S6 the ion remains tightly bound with the binding site distances narrowly distributed around 2.5 Å.

The MCCE2 method^68^ was used to analyze ion binding as a way of determining small-molecule affinity. MCCE2 is a Monte Carlo (MC) type method that uses Boltzmann statistics. The interactions considered between the protein and the ligand (Ca^2^^+^) include a combination of molecular mechanics non-electrostatic interactions with Poisson–Boltzmann^69^ Continuum Electrostatics interactions. The binding affinity is determined by grand canonical Monte Carlo (GCMC) sampling. This approach is well suited to the study of binding, as it allows both the bound and free Ca^2^^+^ ions to reach equilibrium^70^. MCCE samples multi-conformation on the side chains (flexible) within a rigid backbone. The conformer distribution is then determined according to the Poisson–Boltzmann electrostatic interactions, ligand solvation energies, full AMBER^71^ Lennard–Jones, torsion energies, and the solvent-accessible surface area (SAS)-based non-electrostatic ligand–solvent interaction energy^70^. Supplementary Fig. 5 shows the result of Ca^2^^+^-binding affinity simulations. The calculations were carried out exclusively on the activation core portion of the RyR1 (residue B3614–residue B5037). Binding energies were calculated by making the ligand (Ca^2^^+^) compete against a dummy atom (Ca^2^^+^ dummy) in solution.

### Distribution of discrete cluster snapshots on energy landscapes

Supplementary Fig. 6 shows the distribution of snapshots from each of the discrete clusters identified by RELION 3D classification on the energy landscapes. Each closed curve encloses a region densely populated with the snapshots assigned to a discrete structure by RELION. The closed nature of each region is due to the application of a density threshold. Clustering was performed as described in ref. ^22^. The two RyR1 data sets, with and without ligands, were previously clustered into 16 discrete conformational classes by RELION, two of which were classified as “junk”. Class 2 (no ligands) and class 3 (with ligands) are approximately near the extremes of the conformational range observed in this classification. The snapshots emanate from, and are approximately similar to the functionally relevant ligand-free closed and ligand-bound open states described in ref. ^22^.

### Estimating the spatial resolution of the density maps

The procedure for comparing independent half-set reconstructions via Fourier shell correlation is known as the “gold standard” in resolution estimation. This approach cannot be readily used to estimate the resolution of our maps, because the division of the data into two subsets at the outset reduces the conformational sampling to a level incompatible with reliable analysis. The number of available snapshots is already a limiting factor in our analysis, as evidenced by the need to define an orientational aperture radius four times the size commensurate with the 0.4-nm resolution of the data, as estimated by RELION. The division of the data at later points along the analytical pipeline does not produce independent data sets.

We therefore use the following two alternative means to estimate the resolution of our density maps. The program ResMap^72^ estimates the local resolution as ranging from 0.35 nm in the core to 1.2 nm at the outer edge of the C4 symmetrized maps. Structural features in the map core evidently correspond to a resolution of ~0.4 nm, with bulky side chains visible in the best parts of the map (Supplementary Fig. 8). The resolution in the outer parts of the map is ~1.2 nm. This is in large part due to the coarse angular sampling of the data, which limits the resolution in a radius-dependent manner. The original RELION analysis reached ~0.4-nm resolution in the core, similar to the value observed here in the innermost parts of the molecule.

In addition, we measured the resolution reached by RELION refinement of the raw snapshots for all 50 frames of the functional movie. The best achievable resolution was 7.1 Å from a 3000 snapshots subset along the transition path.

### Fitting and refinement of atomic coordinates

Each of the 50 maps along the minimum-energy path was fitted to a model domain by domain with the rigid-body fit function in COOT^73^, using multiple starting models to avoid model bias (PDB ID: 5TB4, 5T9R, 5TAP, 5T9V, 5TAL, 5TAQ)^22^. The models were then refined in real-space using phenix.real_space_refine^74^.

Distance measurements between residue pairs for each of the 50 maps were performed with UCSF Chimera^75^ using residue backbones as references. The distances obtained from different starting models were then averaged. The error bars show the full scatter (not standard deviation) of the results obtained with different starting models.

### Computational resources

All computations were performed on a CPU cluster with the following specifications: 16 CPU nodes, each consisting of two Deca-core E5-2660 V3 “Haswell”/2.6 GHz, and 128 GB of memory. Particle picking, contrast transfer function (CTF) estimation, and initial orientation recovery were performed using RELION. 3D back-projection on 2D NLSA snapshots was executed using the reconstruct function in RELION. UCSF Chimera was used for visualization and compilation of 3D movies^75^. MD simulations were performed using the Summit supercomputer at Oak Ridge Leadership Computing Facility.

### Reporting summary

Further information on research design is available in the Nature Research Reporting Summary linked to this article.

## Supplementary information

Supplementary Information

Description of Additional Supplementary Information

Supplementary Movie 1

Supplementary Movie 2

Supplementary Movie 3

Supplementary Movie 4

Supplementary Movie 5

Supplementary Movie 6

Supplementary Movie 7

Supplementary Movie 8

Supplementary Movie 9

Supplementary Movie 10

Supplementary Movie 11

Supplementary Movie 12

Supplementary Movie 13

Reporting Summary

## Data Availability

The data used in this paper are available at the Protein Data Bank (PDB) in Europe (https://www.ebi.ac.uk/pdbe/) under accession codes 5TB4, 5T9R, 5TAP, 5T9V, 5TAL, and 5TAQ. The cryo-EM density maps for states S1 to S6 have been deposited in the Electron Microscopy Data Bank (EMDB) under accession codes: EMD-20486, EMD-22393, EMD-22395, EMD-22394, EMD-22396, and EMD-22392, and the respective model coordinates have been deposited in the PDB under accession codes 6PV6, 7JMG, 7JMI, 7JMH, 7JMJ, and 7JMF. Particle images used in this study have been deposited in the Electron Microscopy Public Image Archive (EMPIAR) under the accession code EMPIAR-10315. Cryo-EM density maps and models for all 50 states are available from the corresponding authors by request. Additional supporting information regarding the calculations for the binding affinity of calcium ion in RYR1 using MCCE2 can be found at https://github.com/SalahBioPhysics/binding_affinity_ryr1.git. Other data are available from the corresponding authors upon reasonable request.

## Code availability

The software package is available in the following link: https://github.com/GMashayekhi/ManifoldEM_Matlab.git.
