# Supplementary material for: Retrieving functional pathways of biomolecules from single-particle snapshots
Source: Nat Commun. 2020 Sep 18;11:4734. doi: 10.1038/s41467-020-18403-x (PMC7501871; doi:10.1038/s41467-020-18403-x)
Supplement: Supplementary file 1 — Supplementary Information [file 41467_2020_18403_MOESM1_ESM.pdf]

# Supplementary Information

# Retrieving Functional Pathways of Biomolecules from Single-particle Snapshots

A. Dashti, G. Mashayekhi et al.

## Table of Contents

***Supplementary Methods: Verification of data-analytical approach with synthetic data .......... 1***

***Supplementary Note 1: Nature of conformational changes along conformational coordinates 3***

***Supplementary Note 2: Nature of conformational changes along a ligand-binding trajectory . 3***

***Supplementary Note 3: Free energy calculations by molecular dynamics ............................... 4***

***Supplementary References ......................................................................................... 4***

## Supplementary Methods: Verification of data-analytical approach with synthetic data

Here, we demonstrate the ability of our approach to extract the correct conformational movies and energy landscapes from synthetic cryo-EM snapshots. For this purpose, a ribosome-like model with two degrees of freedom entailing rotations of the small subunit about two different axes, was simulated (Supplementary Fig.14 a). Synthetic cryo-EM snapshots of different conformations were generated with probabilities reflecting a conformational energy landscape consisting of a rectangular 3x4 array of 12 energy wells of different depths (Supplementary Fig.14 b). Pixel noise was incorporated via a Gaussian model, with the level of noise adjusted to approximate the experimental values obtained for RyR through the number and distribution of snapshots over the energy landscape. Synthetic snapshots with and without noise were generated for 180 uniformly-spaced projection directions covering a great circle.

In summary, Supplementary Movies 10 and 11 show the conformational variations in the input synthetic model, viewed in a typical projection direction, by sorting the snapshots based on the rotation angle 1 (Supplementary Movie 10) and rotation angle 2 (Supplementary Movie 11). The conformational motions are complex, due to the combined operation of two different subunit rotations over an undulating energy landscape. Supplementary Movies 12 and 13 demonstrate that our analytical pipeline separates these apparently complex motions

into the two conformational motions used to generate the synthetic model. The energy landscape obtained by our analysis reproduces the topologically correct energy landscape, albeit with respect to a different metric. (Supplementary Fig. 16). We note that the choice of a conformational metric is arbitrary.

We now outline the outcome of each major step in the analytical pipeline. Please also see previous publications [1] and [2] and references therein, where the analytical pipeline and its application to synthetic data with and without noise, and with and without defocus variations are described.

a. **Determining the conformational manifold in each projection direction**
   We use diffusion-map embedding of the snapshots in each projection direction to determine the conformational manifold. The two conformational coordinates selected by the algorithm are used to represent the manifold (Supplementary Fig. 15). The performance of the defocus-tolerant kernel with noisy and noise-free synthetic data is described in detail in [1].

b. **Nonlinear Laplacian Spectral Analysis (NLSA)[3] along eigenfunctions for each projection direction**
   This step visualizes the conformational changes along each diffusion map eigenfunction (conformational coordinate). This information can be used to identify biologically interesting conformational coordinates. Supplementary Movies 12 and 13 represent the conformational movies along the first two eigenfunctions ranked according to eigenvalue.

c. **Propagating the conformational coordinate among projection directions**
   The order of the eigenfunctions ranked according to eigenvalue may be different in different projection directions, because some conformational changes appear stronger in some viewing angles. Also, the sense of the conformational changes may change due to sense-neutrality in eigen-decomposition. In order to establish a consistent set of conformational coordinates across different projection directions, we use a Nyström extension scheme [4] to describe a common subset of snapshots in terms of the manifolds from each of two neighboring projection directions. This allows us to determine the affine transformation needed to transform a given manifold to that of a neighboring projection direction.

d. **Metric homogenization across projection directions**
   The manifold in each projection direction is characterized by a so-called induced metric (local measure of distance), which is, in general, non-uniform (inhomogeneous) over each manifold, and can change from projection direction to projection direction. We solve this problem as described in detail in[1]. In brief, we use NLSA to map the manifold to a space of known eigenfunctions. In each projection direction, the snapshots are ordered according to their projections on the family of straight lines through the origin, each making a different angle $\theta$ with the horizontal axis. The snapshots are then concatenated along each line to form supervectors. By performing NLSA on the supervectors, we extract the conformational evolution governed by the conformational parameter $\tau$ along the selected line characterized by $\theta$. Given a sufficiently dense

collection of radial lines, the conformational changes can be described in any direction in the multidimensional space of conformations by the parameter $\tau(\theta)$. The mapping from a space of unknown metric to one characterized by a parameter $\tau(\theta)$ allows a consistent description of the conformational changes among the projection directions. We note that the choice of the conformational metric is arbitrary.

e. **Mapping the multi-dimensional energy landscape**
Armed with a universal description for conformational changes along each radial line over the manifolds from all the projection directions, we use tomographic reconstruction (inverse Radon transform with the Shepp-Logan filter) to determine the 2D landscape compiled from all projection directions along a great circle. In Supplementary Fig.14b and c, the occupancy map produced by the algorithm is shown next to the input landscape. Although the latter is in terms of a metric different from that used to generate the synthetic data, it preserves the topology of the input landscape, thus allowing identification of the correct least-action trajectory. Supplementary Fig. 16 shows that the distribution of snapshots is regionally well preserved by the algorithm, with and without pixel noise, even when noise masks the variations in the input snapshot distribution (Supplementary Fig. 15 b). Snapshots belonging to a specific region of the input landscape are mapped to the equivalent region of the output landscape. Taken together, these provide strong support for the veracity of our approach.

## Supplementary Note 1: Nature of conformational changes along conformational coordinates

Here, we show the nature of conformational changes along each of the two conformational coordinates identified by our analysis. NLSA (singular value decomposition on a curved manifold) [3] with snapshots ordered according to their projection on a given conformational coordinate reveals the evolution of the data along that conformational coordinate. Using the snapshots in one projection direction, Supplementary Fig. 5 shows the conformational deviation from the mean along the two most important conformational coordinates CC1 and CC2. The other above-noise eigenfunctions of the Diffusion Map manifold reveals residual artifact motions stemming primarily from a cluster of snapshots with exceptionally low contrast (see Supplementary Fig. 9). Supplementary Movie 9 represents the movements observed along higher eigenfunctions of the manifold.

## Supplementary Note 2: Nature of conformational changes along a ligand-binding trajectory

Maps and models compiled along the functional ligand-binding trajectory show domain motions, such as up-down shell movement, rotation of the activation domain, pore opening, and rotation of the pseudo-voltage sensor domain (pVSD). The concerted nature of these motions may be the result of direct coupling between the respective domains (Figs. 2 - 5, Supplementary Figs. 10 and 11). As these figures show, it is not possible reliably to capture complex transitory motions by morphing between two discrete RELION structures, via, e.g., the "Morph Conformations"

facility in Chimera
(https://www.cgl.ucsf.edu/chimera/docs/ContributedSoftware/morph/morph.html).

Supplementary Fig. 12 and 13 show the binding pocket of ATP and $Ca^{2+}$ in the vicinity of the transition "hotspot". As the figures show, transition to the lower (with-ligands) landscape initiates the appearance of a partly-bound ATP, ending in full binding at the energy minimum on the lower landscape. This observation indicates an increase in the fraction of RyR1 bound to ATP as the minimum-energy point on the +ligand (lower) landscape is approached.

## Supplementary Note 3: Free energy calculations by molecular dynamics

In order to describe the complex conformational changes between the conformational states S1 and S6, path collective variables [5] were employed. Subsequently, multiple walker eABF was used as an importance sampling [6] algorithm to describe the free energy associated with the conformational changes (Supplementary Figs. 7-9). In order to describe the path collective variable, a reference path was defined by a set of conformations, including only the $C_\alpha$ and $C_\beta$ atoms of the $Ca^{+2}$ binding site from the six cryo-EM structures. Progress along the path is described mathematically as follows :

$$S_{path}(r) = \frac{\sum_{i=1}^{N} i \exp^{-\lambda M_i(r)}}{\sum_{i=1}^{N} \exp^{-\lambda M_i(r)}} \quad (1)$$

Here $r$ is the instantaneous protein conformation sampled in an MD simulation, $N$ the number of reference conformations describing the path (here $N$=6). $M_i$ is the mean-square deviation between the sampled and the $i$th reference conformation, λ is a smoothing factor, whose value is the inverse of mean-square deviation. [6]

The auxiliary collective variable can be described mathematically as follows:

$$Z_{path}(r) = \frac{-1}{\lambda} \ln \sum_{i=1}^{N} i \exp^{-\lambda M_i(r)} \quad (2)$$

Application of the auxiliary collective variable ensures that the trajectories sample the regions of conformational space corresponding to a tube of radius 2.5Å. This radius represents the maximum deviation between any two structures from the MD trajectories, as presented in Supplementary Fig. 2. Consequently, a diverse ensemble of structures is sampled during the free energy calculation. Using the six conformations as six independent walkers, free energy along the path was obtained for both the apo and $Ca^{+2}$ bound cases by performing eABF simulations. In these simulations, the samples were exchanged every 10ps and the biasing forces were applied after collecting 5,000 force samples. Each walker was simulated for around 350 ns, resulting in a cumulative sampling of >2.0 microseconds. Supplementary Fig. 1 shows the results of free-energy calculations along the states S1-S6. The error bars are calculated as standard deviation of the free energy obtained from the last 50 ns of the eABF calculation for every window. The narrow distribution of the error (accumulated over 6 x 50 ns = 300 ns) relative to the mean values of the free-energy features support convergence.

## Supplementary References

1. Dashti, A. et al. Supporting Information:Trajectories of the ribosome as a Brownian nanomachine. *Proc Natl Acad Sci U S A* **111**, 17492-7 (2014).
2. Schwander, P., Fung, R. & Ourmazd, A. Conformations of macromolecules and their complexes from heterogeneous datasets. *Phil Trans R Soc B* **369**, 20130567 (2014).
3. Giannakis, D. & Majda, A.J. Nonlinear Laplacian spectral analysis for time series with intermittency and low-frequency variability. *Proc Natl Acad Sci U S A* **109**, 2222-2227 (2012).
4. Lafon, S., Keller, Y. & Coifman, R.R. Data fusion and multicue data matching by diffusion maps. *IEEE Trans Pattern Anal Mach Intell* **28**, 1784-1797 (2006).
5. Branduardi, D., Gervasio, F.L. & Parrinello, M. From A to B in free energy space. *J Chem Physics* **126** (2007).
6. FU, H., Shao, X., Chipot, C. & Cai, W. Extended Adaptive Biasing Force Algorithm. An On-the-Fly Implementation for Accurate Free-Energy Calculations. *J. Chem. Theory Comput*, 3506-3513 (2016).

## Supplementary Figures

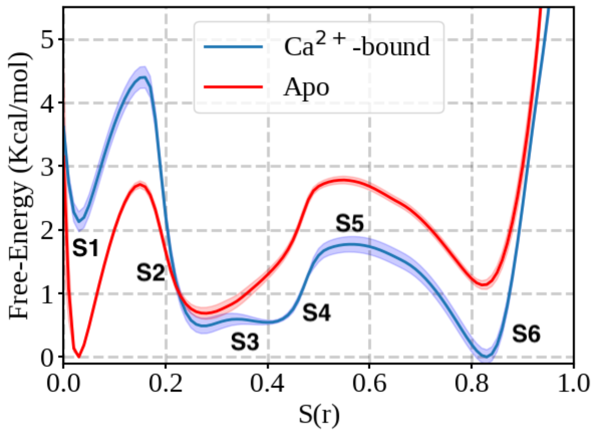


**a**

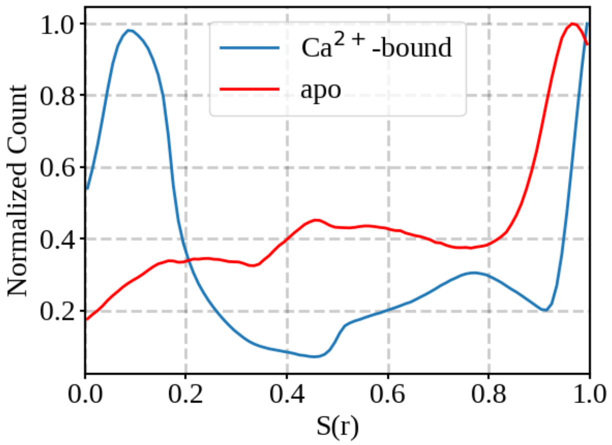


**b**

**Supplementary Fig. 1. Energy and sample distribution along the path collective variable S(r).**

**a.** Free-energy landscape of the $Ca^{+2}$ ion bound and the apo state of the binding pocket moving from an open (S=0) to a closed conformation (S=1). The path collective variable is a function of the instantaneous positions of the ion and GLU3697, E3771 and THR5001. The states S1 to S6 are located along the path of Fig. 1a in the main text. Moving from S1 to S6 is energetically uphill in the apo-pocket, with no $Ca^{+2}$ bound. In contrast, with the ion present in the vicinity of the pocket, the S1 to S6 transition is spontaneous. Also note that, in S1 and S2 conformations, the apo state is more stable than the bound, indicating that ion-binding is implausible. Consistent with the association energetics of Supplementary Fig. 5, binding proceeds from state S3 onwards.

**b.** Normalized counts representing the samples accrued in each bin along the path variable S(r), in the multiple-walker eABF simulation. The full (0 - 1) range of S(r) was discretized into 100 bins of equal width. Blue and red traces represent the counts for the $Ca^{+2}$ bound, and apo systems, respectively.

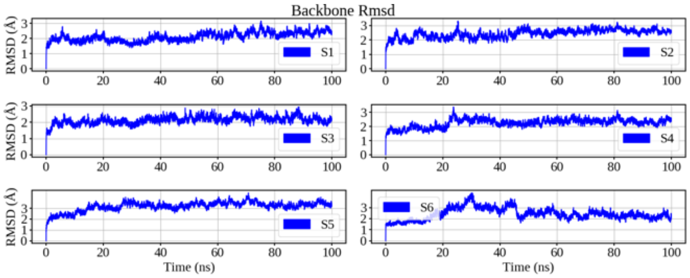


**a**

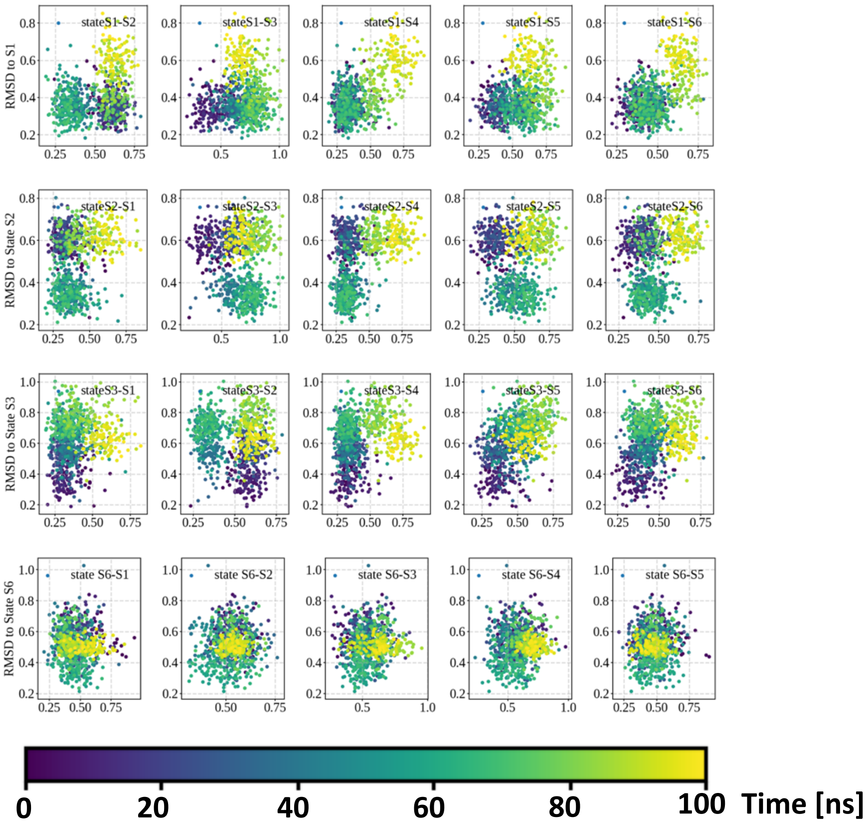


**b**

**Supplementary Fig. 2. Stability of MD models.**

- **a.** Time trace of the root mean square deviation (RMSD) of the backbone atoms of the $Ca^{2+}$ binding domain from the initial structures. The simulations were initiated from states S1-S6. The states S1 to S6 are located along the path of Fig. 1a in the main text.
- **b.** Frame-by-frame RMSD matrix across 100 ns of MD simulations computed with respect to state S1 (row 1), S2 (row 2), S3 (row 3), S4 (row 4) and S5 (row 5), showing minor changes in the binding pocket.

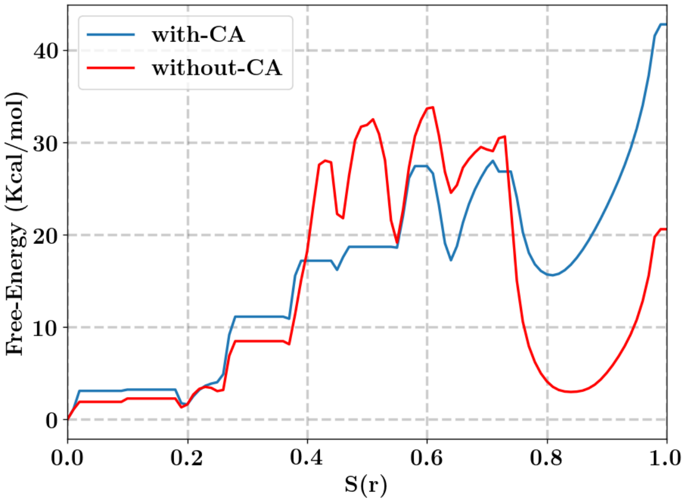


**Supplementary Fig. 3. Free energy profile of the $Ca^{+2}$ bound and apo state of the binding pocket from an open (S=0) to a closed conformation (S=1).** The end states were obtained from the cryo-EM structures. Steered molecular dynamics was utilized to extract the intermediate states. The path collective variable is a function of the instantaneous positions of the ion and GLU3697, E3771 and THR5001. The states S1 to S6 are located along the path of Fig. 1a in the main text. Moving from S1 to S6 is energetically uphill in both the apo, and the $Ca^{+2}$ bound states, with an large ~30 kcal/mol energetic barrier between the states. This energetic barrier points to the suboptimal path obtained from the steered MD simulations, highlighting the need for experimental i.e. manifold-derived intermediate states to enhance the quality of the transition pathway.

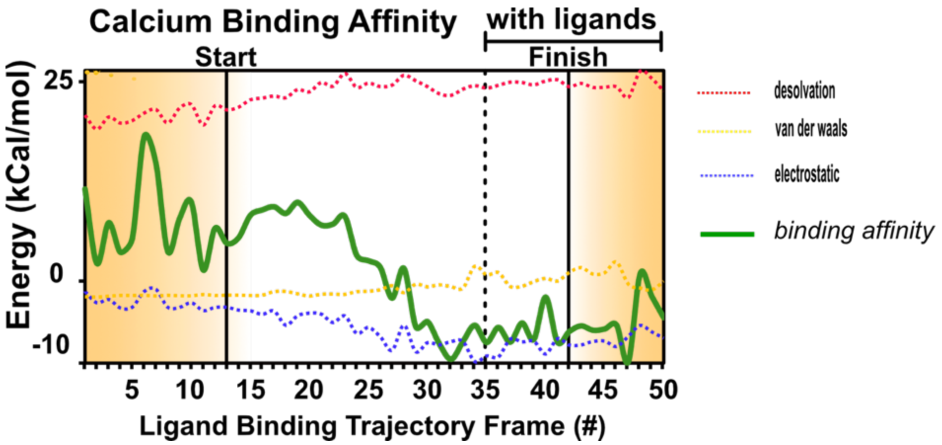


**Supplementary Fig. 4. The binding affinity (green) of $Ca^{+2}$ in RYR1.** The energy contributions of desolvation (red), Van der Waals (yellow), and electrostatic effects (blue) are also shown. The measurements are plotted as described in Fig. 2 of the paper. The binding energy of the $Ca^{+2}$ (green) turns negative near the transition (HOT) region of Figs. 1a and b, which indicates greater favorability for $Ca^{+2}$ binding.

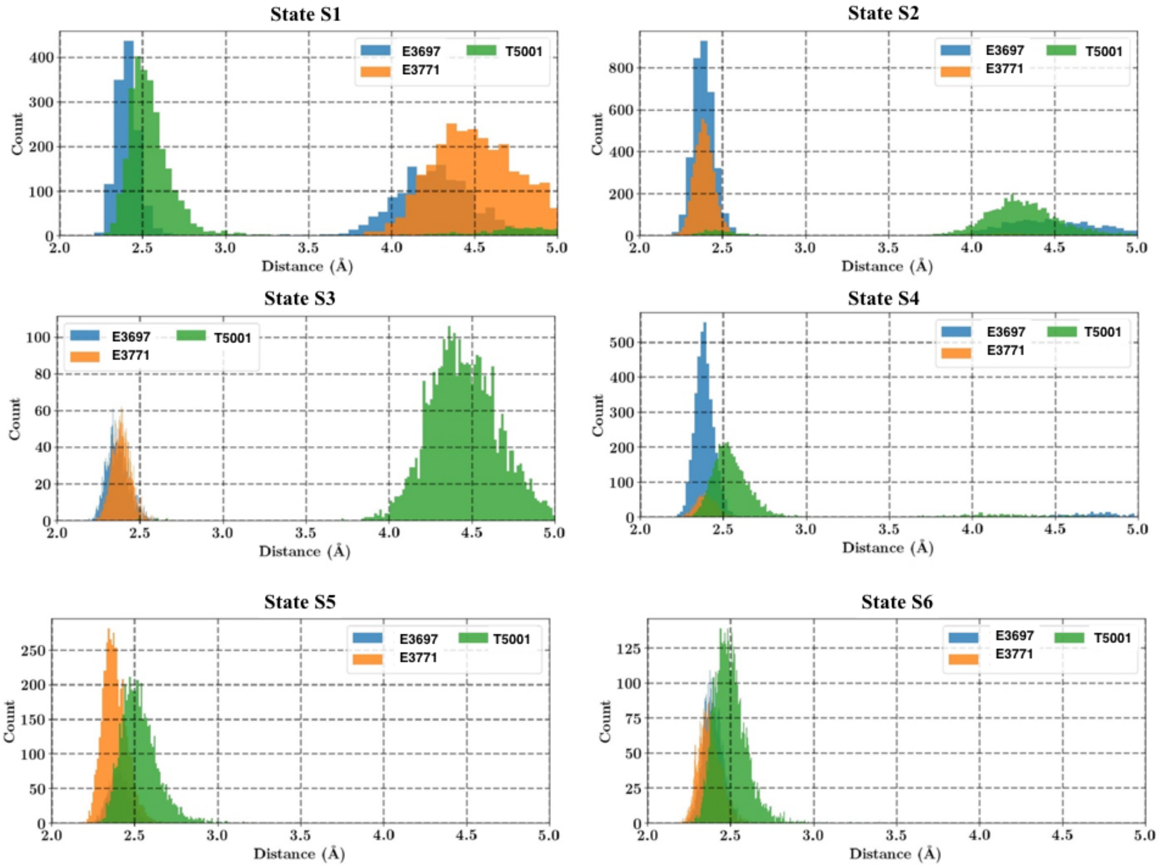


**Supplementary Fig. 5. Distance distribution of the $Ca^{+2}$ ion relative to the binding pocket GLU and THR residues across states S1 to S6.** Each state was simulated to equilibrium in 100-ns MD simulations. In S1, the $Ca^{+2}$ distances from GLU3697 and THR5001 form a bimodal distribution, and E3771 engages only weakly with the ion. The ion associates completely with the pocket in state S4 and remains associated in S5 and S6.

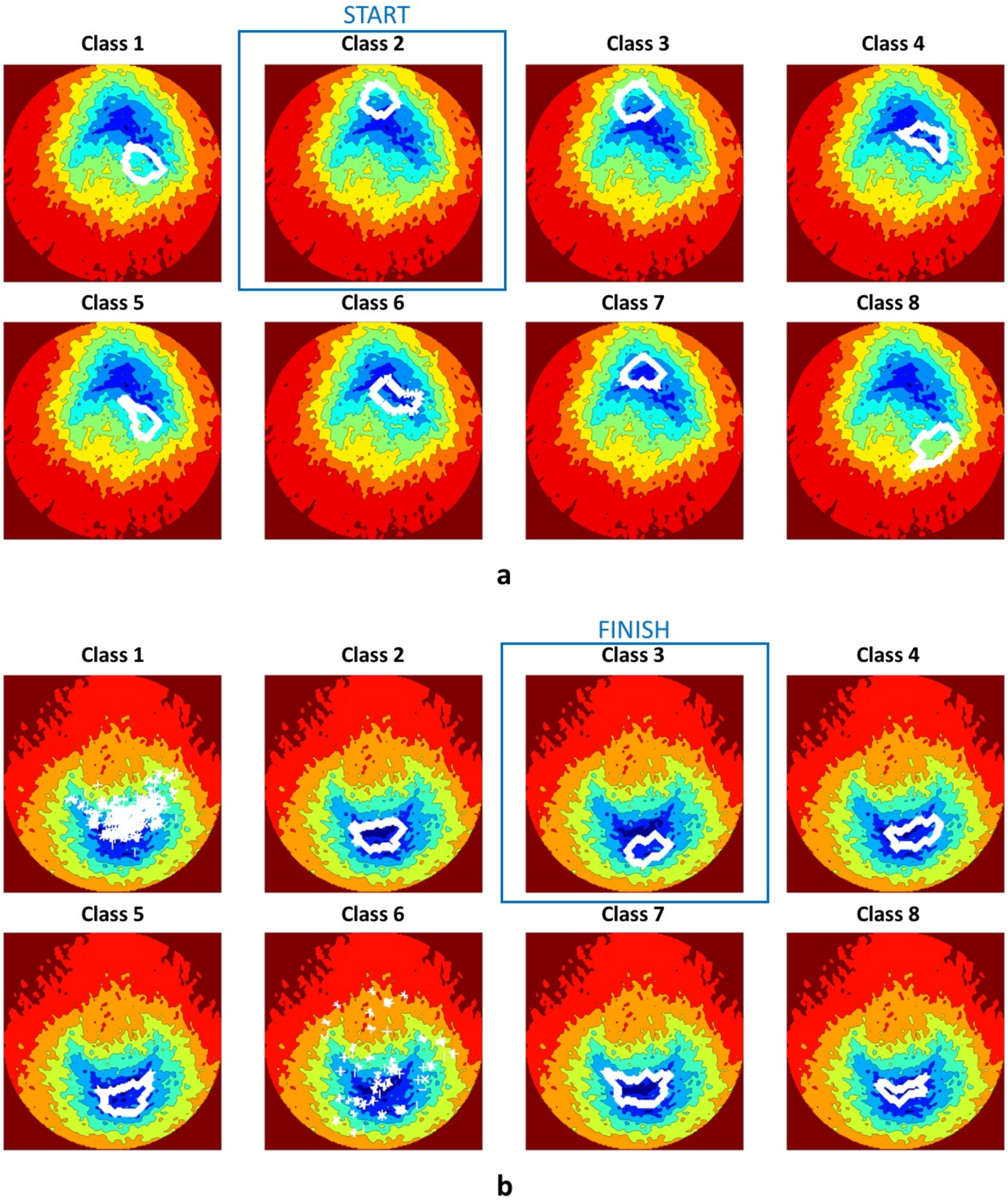


**Supplementary Fig. 6. Distribution of snapshots from each discrete cluster on the energy landscapes of Fig. 1a.**

a. No ligands.
b. With ligands.

The boxed classes were previously identified as the extremes of the conformational range and used to infer function by discrete cluster analysis.

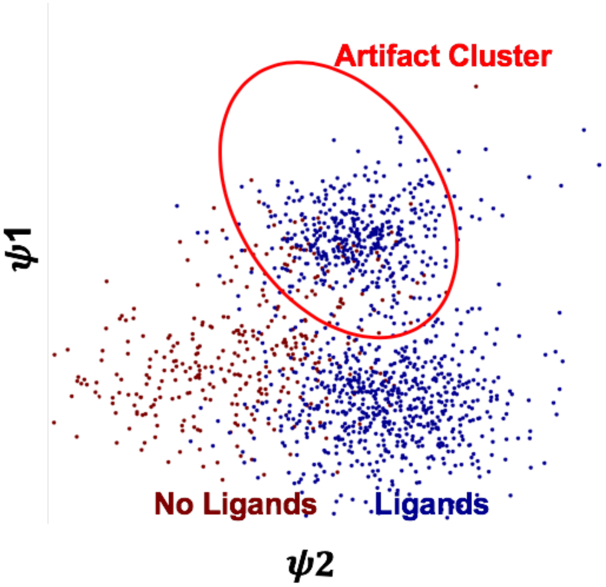


**a**

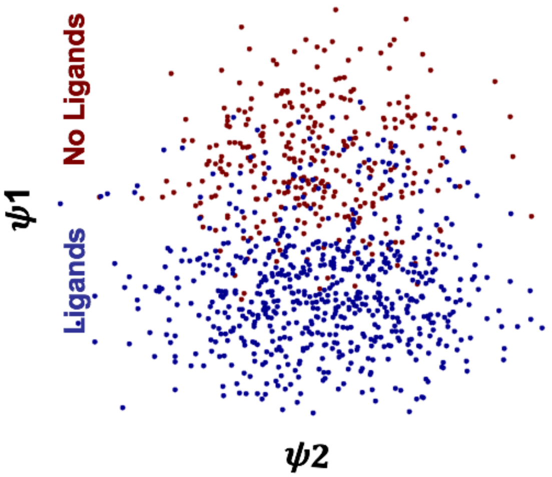


**b**

**Supplementary Fig. 7. Manifold-based preprocessing of data.**

**a.** The initial manifold RyR1 snapshots in one projection direction. The points are color-coded based on the -/+ligand subset they are originated from. The manifold reveals two clusters. One of the clusters ("Artifact") contains snapshots of unusually low contrast. These snapshots were removed before further processing.

**b.** The data manifold after excluding the snapshots belonging to the artifact cluster.

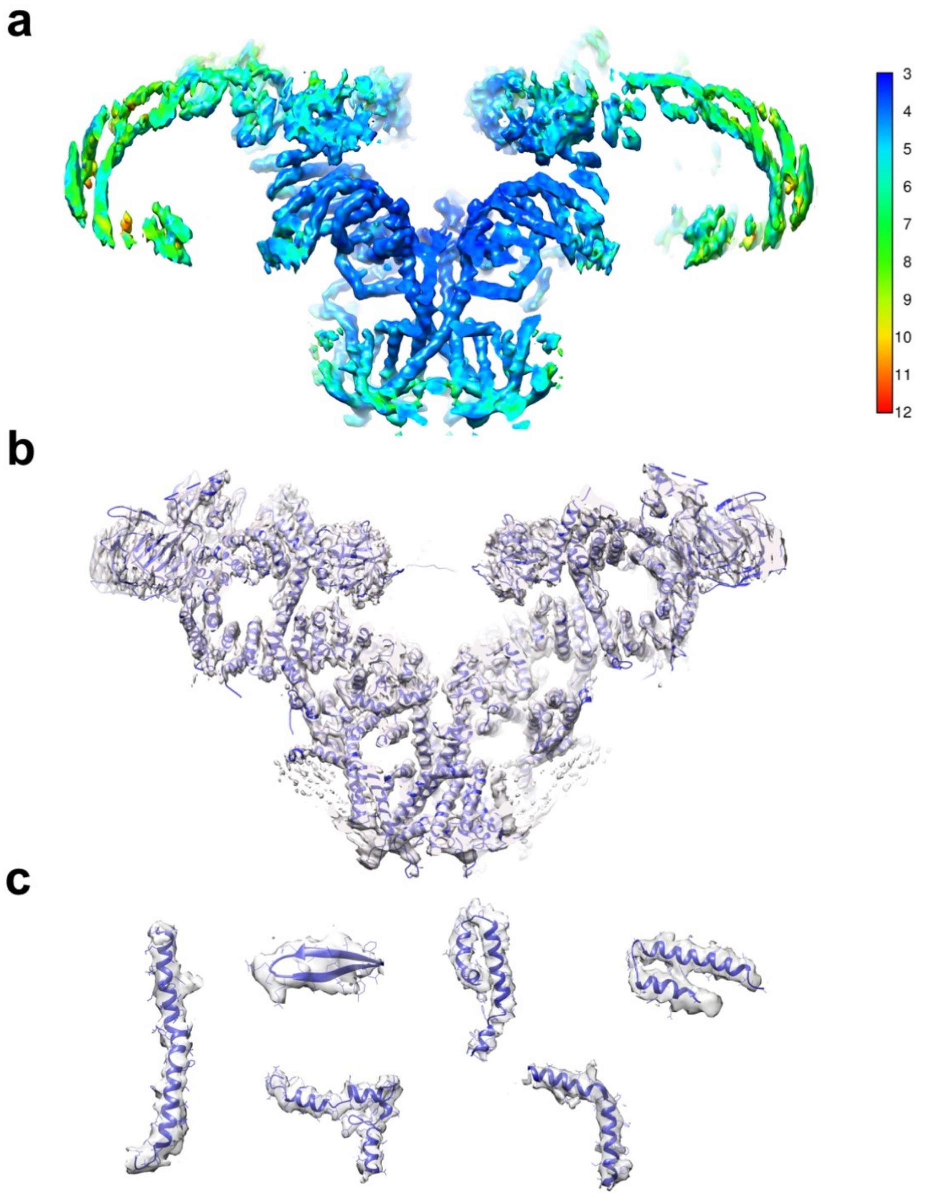


**Supplementary Fig. 8. Spatial resolution.**

a. Slab through one of the cryo-EM reconstructions of RyR1 along the trajectory, colored according to the local resolution as measured by ResMap (color bar in 0.1nm units).
b. Slab through the same cryo-EM map fitted with the atomic model of RyR1. Map and model are shown in transparent surface and ribbon representations, respectively.
c. Densities and models of subsets of secondary structure elements in different regions of the map.

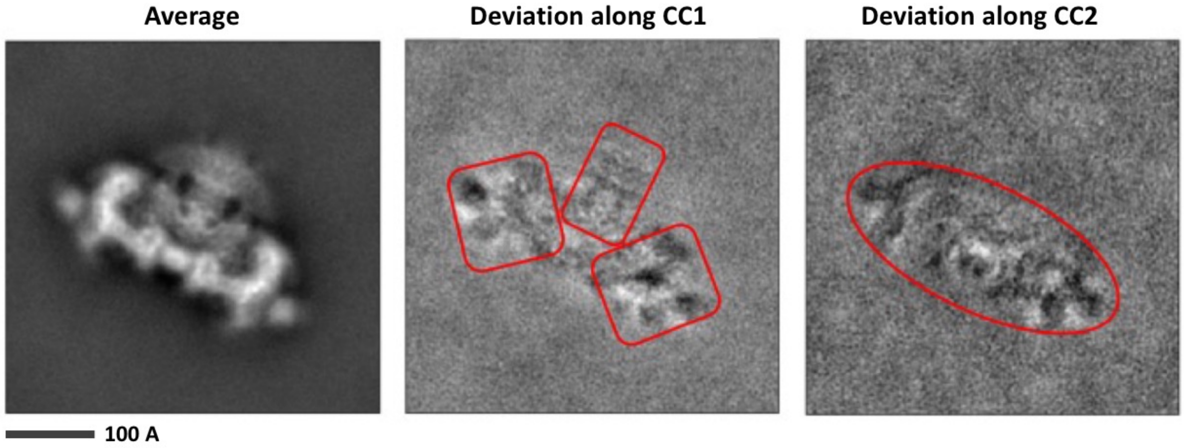


**Supplementary Fig. 9. Average structure and deviations from it along the two primary conformational coordinates CC1 & CC2**. Viewed in one sample projection direction. The deviations from the mean reveal the concerted conformational changes associated with each conformational coordinate. Corresponding changes are observed in all projection directions.

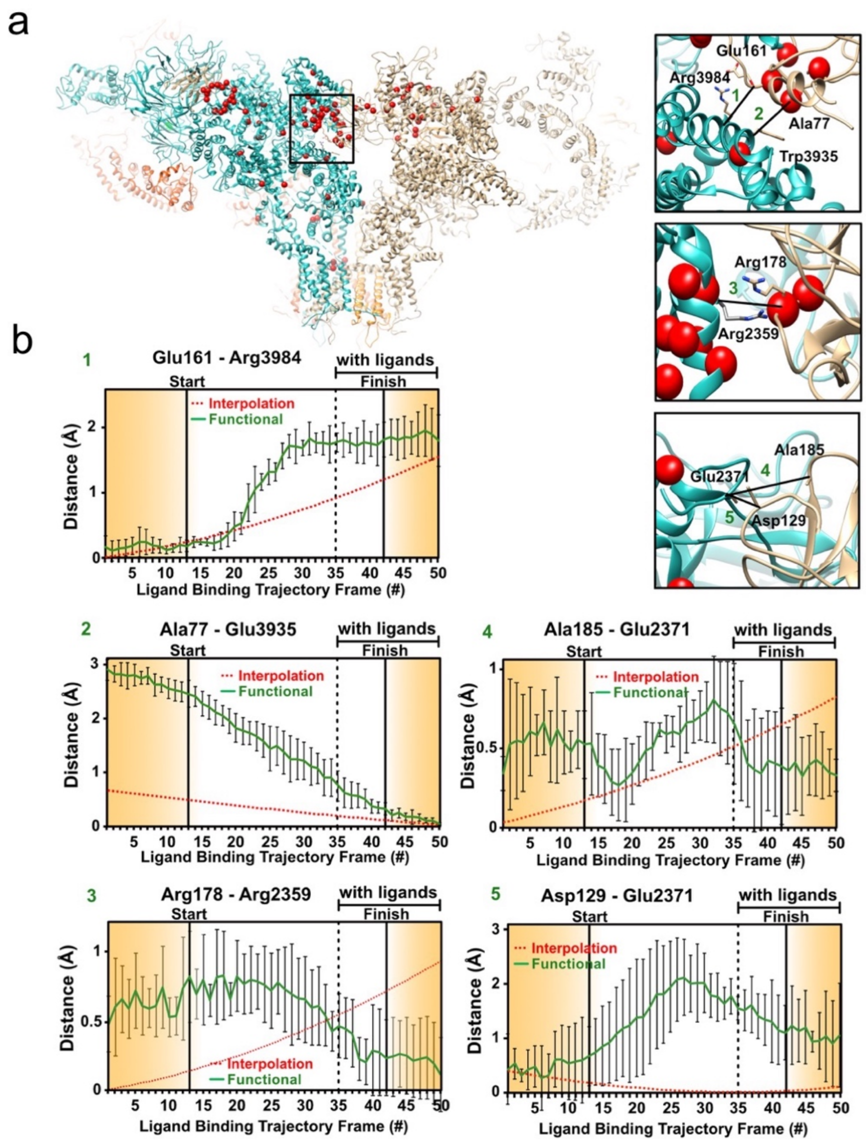


**Supplementary Fig. 10. Changes at interprotomer contact sites in N-terminal domain along the route of Fig. 1a,** augmented with excursions from the minimum-energy points along conformational coordinate 1.

a. The general region, and the specific sites examined in detail, with each monomer shown in different colors. The locations of known point-mutations found in malignant hyperthermia 1 and central core disease of muscle are shown in red.
b. Distance variations between carbon-alpha backbone atoms of two opposing residues for each of the frames along the functional trajectory. The measurements were calculated and plotted as described in Fig. 2 of the main text. Measured distances between two amino acid backbones at contact sites between the monomers. The amino acids used for measurement are represented as sticks. Error bars represent the full scatter (not standard deviation) of the results obtained with 6 different starting models.

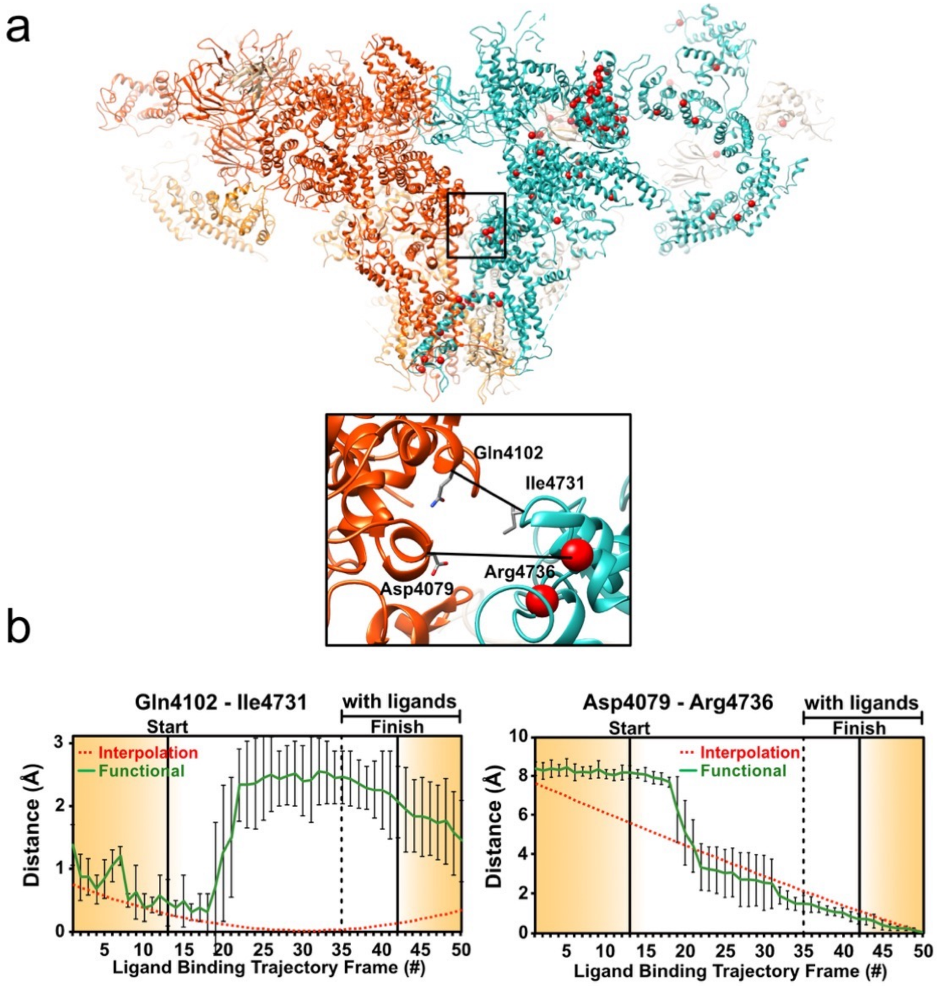


**Supplementary Fig. 11. Changes at interprotomer contact sites in the activation core along the route of Fig. 1a,** augmented with excursions from the minimum-energy points along RC1.

a. The general region and the specific sites examined in detail, with each monomer shown in a different color. The locations of known point-mutations found in malignant hyperthermia 1 and central core disease of muscle are shown in red.
b. Distance variations measured between carbon-alpha backbone atoms of two opposing residues for each of the frames along the functional trajectory. The measurements were calculated and plotted as described in Fig. 2 of the main text. Measured distances are between two amino acid backbones at contact sites between the monomers. The amino acids used for measurement are represented as sticks. Error bars represent the full scatter (not standard deviation) of the results obtained with 6 different starting models.

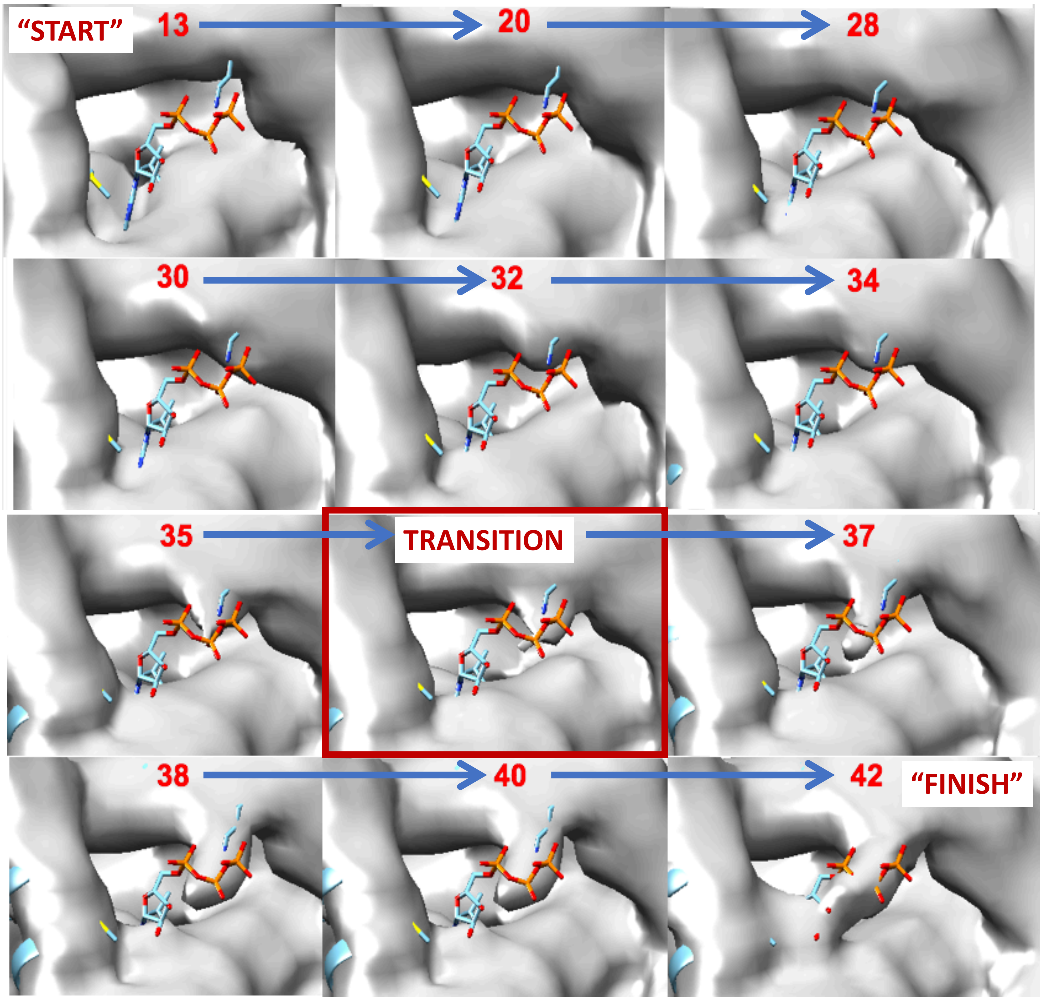


**Supplementary Fig. 12. Changes at the ATP binding site along the functional path of Fig.1a.** Frames before "TRANSITION" stem from the upper no-ligand landscape, the subsequent frames from the lower with-ligand landscape. The START and FINISH labels pertain to the functional trajectory of Fig. 1a of the main text. No or little density is visible in no-ligand frames of the trajectory. Density at the ATP site begins to appear immediately before the transition to the lower landscape, growing as the minimum-energy "FINISH" point is approached, where a fully tethered ATP is observed.

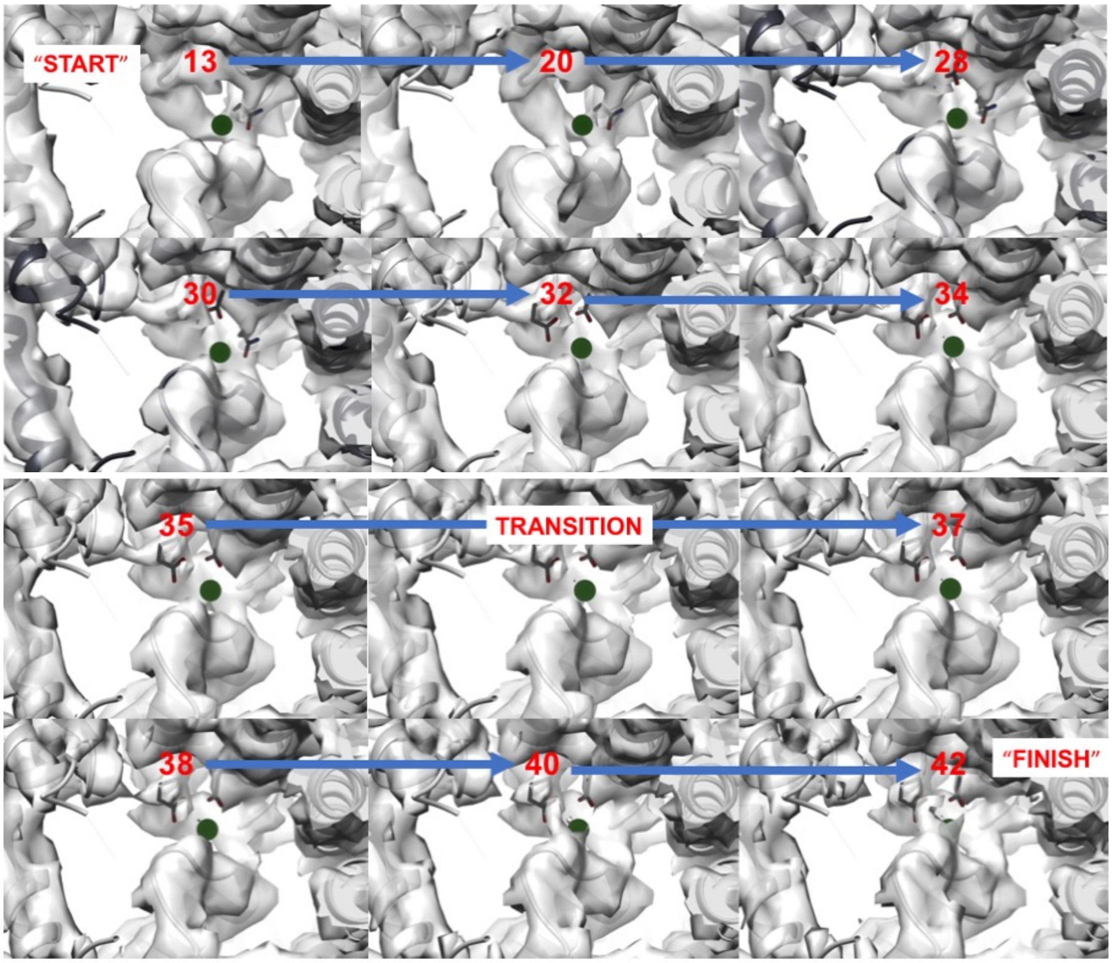


**Supplementary Fig. 13. Changes at the $Ca^{2+}$ binding site along the functional path of Fig.1a.** Frames before "TRANSITION" stem from the upper no-ligand landscape, the subsequent frames from the lower with-ligand landscape. The START and FINISH labels pertain to the functional trajectory of Fig. 1a of the main text. No or little density is visible around the $Ca^{2+}$ (green dot) in no-ligand frames of the trajectory whereas after transition, some density starts to appear.

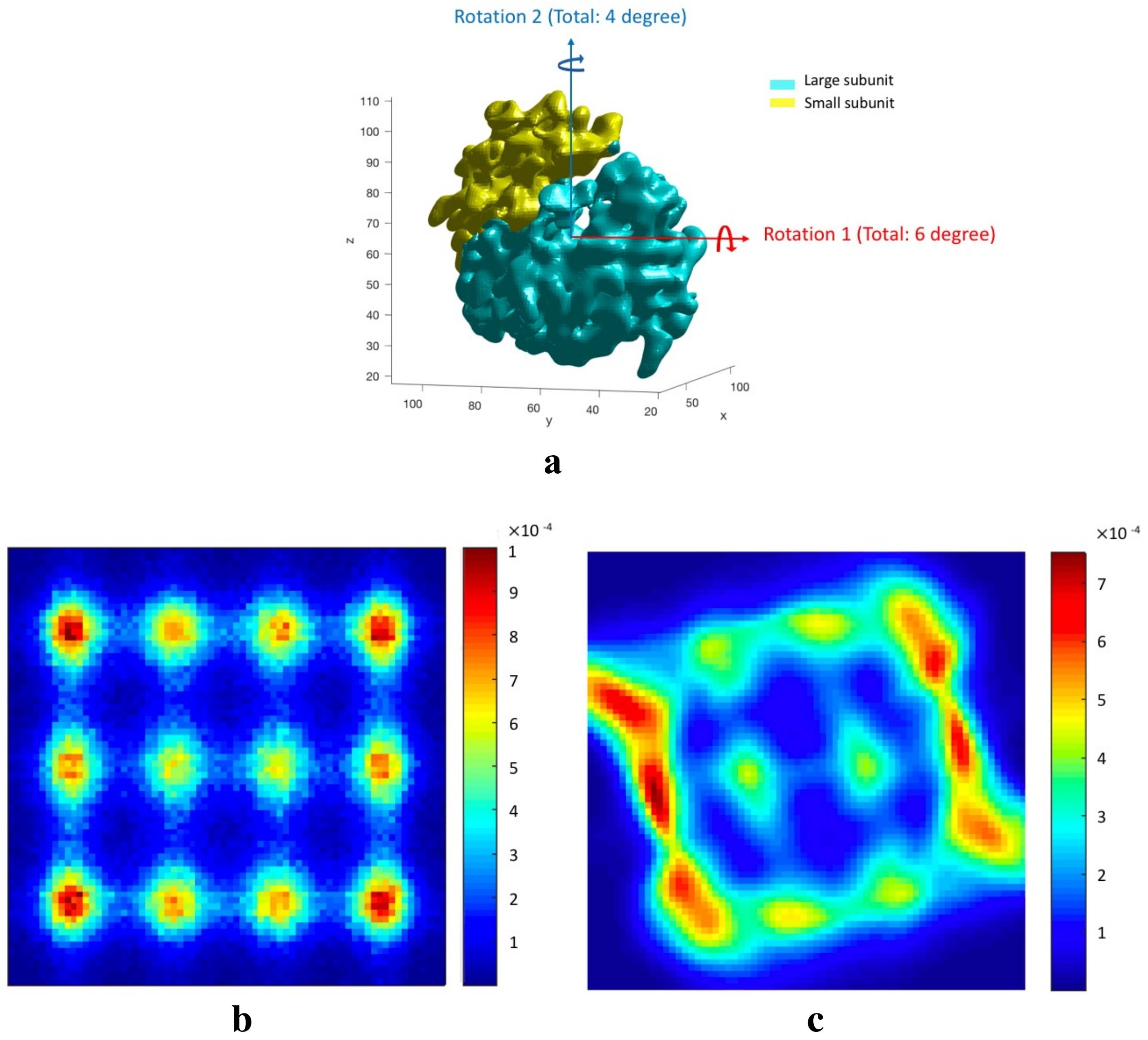


**Supplementary Fig. 14. Synthetic Model**

a. Sketch of the synthetic model, rotation of the small subunit about the two axes
b. Occupancy map used to generate synthetic data.
c. Occupancy map obtained by our algorithm for synthetic data without pixel noise, stemming from all projection directions on a great circle.

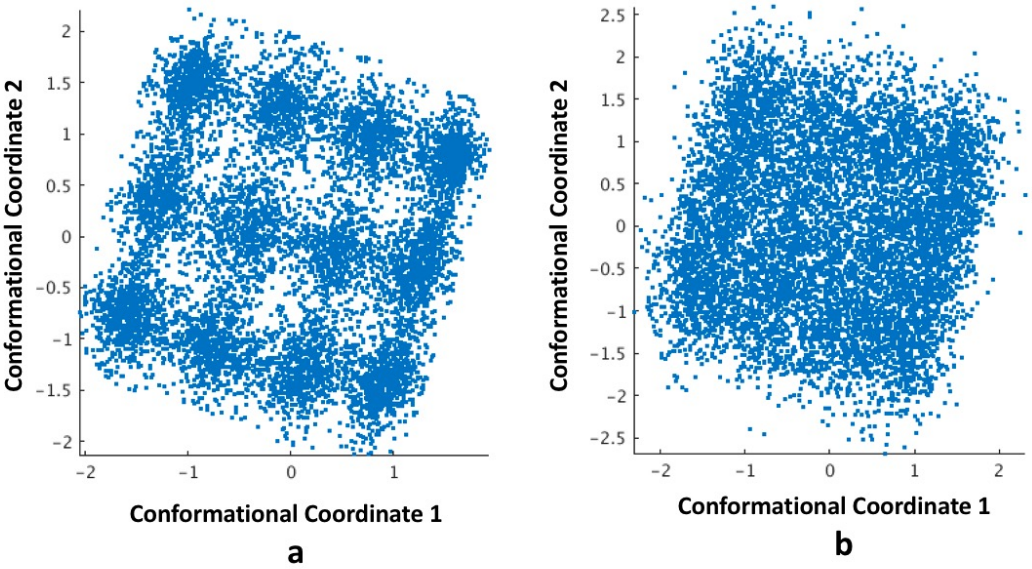


**Supplementary Fig. 15. Manifold of a typical projection direction for the synthetic data with two conformational coordinates.**

a. Without pixel noise
b. With pixel noise.

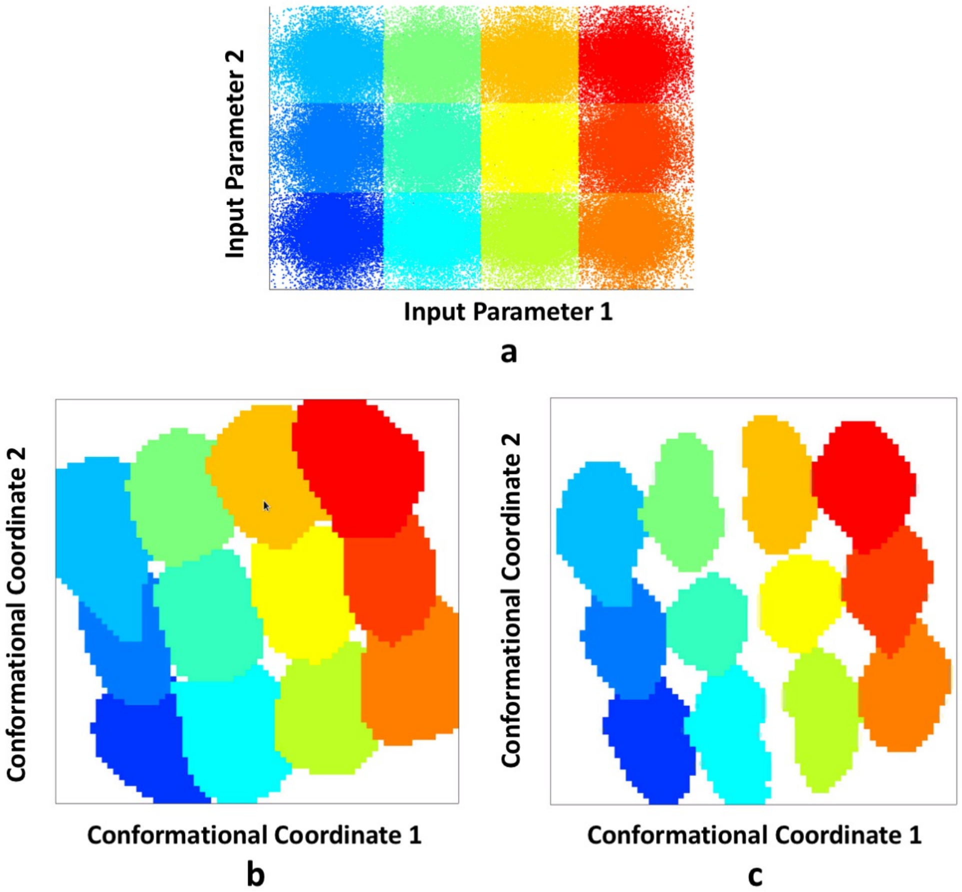


**Supplementary Fig. 16. Parentage plots for comparison of the input and output landscapes**
a. Distribution of snapshots over the input occupancy landscape
b. Distribution obtained by our algorithm, without pixel noise
c. Distribution obtained by our algorithm, with pixel noise.
These maps are obtained as follows. The input landscape is divided into 12 regions each represented by a different color. The snapshots belonging to each of the 12 regions of the input landscape are represented by the same color on the landscape obtained by our algorithm. Note: To facilitate comparison, trivial inversions have been corrected.
