# Supplementary material for: Retrieving functional pathways of biomolecules from single-particle snapshots
Source: Nat Commun. 2020 Sep 18;11:4734. doi: 10.1038/s41467-020-18403-x (PMC7501871; doi:10.1038/s41467-020-18403-x)
Supplement: Supplementary file 2 — Description of Additional Supplementary Information [file 41467_2020_18403_MOESM2_ESM.pdf]

Description of Additional Supplementary Files

File Name: Supplementary Movie 1
Description: Conformational changes along the functional trajectory of Fig. 1a (trans-membrane view).

File Name: Supplementary Movie 2
Description: Conformational changes along the functional trajectory of Fig. 1a (cytoplasmic view).

File Name: Supplementary Movie 3
Description: Conformational changes along the functional trajectory of Fig. 1a represented by the atomic displacements (Color bar shows the magnitude of the atomic displacements)

File Name: Supplementary Movie 4
Description: Conformational changes obtained by linear interpolation between the ligand-free closed and ligand-bound open discrete structures and represented by the atomic displacements. (Color bar shows the magnitude of the atomic displacements).

File Name: Supplementary Movie 5
Description: Conformational changes in the asymmetric unit of the activation core domain along the functional trajectory of Fig. 1a represented by the atomic displacements. Colored spheres indicate ligand-binding sites (yellow: Ca2+; magenta: caffeine; brown: ATP). Color bar shows the magnitude of the atomic displacements.

File Name: Supplementary Movie 6
Description: Conformational changes in the asymmetric unit of the activation core domain obtained by linear interpolation between the ligand-free closed and ligand-bound open discrete structures and represented by the atomic displacements. Colored spheres indicate ligand-binding sites (yellow: Ca2+; magenta: caffeine; brown: ATP). Color bar shows the magnitude of the atomic displacements.

File Name: Supplementary Movie 7
Description: Conformational changes in the Ca+2 binding site in interaction with the Ca+2 ligand in state 1 [“START”] along the functional trajectory of Fig. 1a] coming from MD simulation.

File Name: Supplementary Movie 8
Description: Conformational changes in the Ca+2 binding site in interaction with the Ca+2 ligand in state 6 (“FINISH” along the functional trajectory of Fig. 1a) coming from MD simulation.

File Name: Supplementary Movie 9
Description: The two-dimensional movie along the higher eigenfunctions of the manifold representing an unusual reduction of contrast in some experimental snapshots.

File Name: Supplementary Movie 10
Description: Conformational variations in the input synthetic model, when the snapshots are sorted based on the rotation angle 1, viewed in a typical projection direction.

File Name: Supplementary Movie 11
Description: Conformational variations in the input synthetic model, when the snapshots are sorted based on the rotation angle 2, viewed in a typical projection direction.

File Name: Supplementary Movie 12
Description: Conformational motions along the conformational coordinate 1 compiled by the pipeline for the synthetic data, viewed in a typical projection direction.

File Name: Supplementary Movie 13
Description: Conformational motions along the conformational coordinate 2 compiled by the pipeline for the synthetic data, viewed in a typical projection direction.
