# Supplementary material for: Retrieving functional pathways of biomolecules from single-particle snapshots
Source: Nat Commun. 2020 Sep 18;11:4734. doi: 10.1038/s41467-020-18403-x (PMC7501871; doi:10.1038/s41467-020-18403-x)
Supplement: Supplementary file 16 — Reporting Summary [file 41467_2020_18403_MOESM16_ESM.pdf]

nature research

Corresponding author(s): Abhishek Singharoy, Amedee des Georges,Joachim Frank, Abbas Ourmazd

Last updated by author(s): Aug 14, 2020

Reporting Summary

Nature Research wishes to improve the reproducibility of the work that we publish. This form provides structure for consistency and transparency in reporting. For further information on Nature Research policies, see Authors & Referees and the Editorial Policy Checklist .

Statistics

For all statistical analyses, confirm that the following items are present in the figure legend, table legend, main text, or Methods section.

n/a	Confirmed	
☐	☒	The exact sample size (*n*) for each experimental group/condition, given as a discrete number and unit of measurement
☐	☒	A statement on whether measurements were taken from distinct samples or whether the same sample was measured repeatedly
☐	☒	The statistical test(s) used AND whether they are one- or two-sided<br>*Only common tests should be described solely by name; describe more complex techniques in the Methods section.*
☐	☒	A description of all covariates tested
☐	☒	A description of any assumptions or corrections, such as tests of normality and adjustment for multiple comparisons
☐	☒	A full description of the statistical parameters including central tendency (e.g. means) or other basic estimates (e.g. regression coefficient) AND variation (e.g. standard deviation) or associated estimates of uncertainty (e.g. confidence intervals)
☒	☐	For null hypothesis testing, the test statistic (e.g. *F*, *t*, *r*) with confidence intervals, effect sizes, degrees of freedom and *P* value noted<br>*Give P values as exact values whenever suitable.*
☒	☐	For Bayesian analysis, information on the choice of priors and Markov chain Monte Carlo settings
☐	☒	For hierarchical and complex designs, identification of the appropriate level for tests and full reporting of outcomes
☒	☐	Estimates of effect sizes (e.g. Cohen's *d*, Pearson's *r*), indicating how they were calculated

Our web collection on statistics for biologists contains articles on many of the points above.

Software and code

Policy information about availability of computer code

Data collection	RELION 1.3 ,MotionCorr, CTFFIND4
Data analysis	We developed our own algorithms in MATLAB for data analysis. The algorithms are being released as an open source software called ManifoldEM. We also used following software: UCSF Chimera, ResMap, COOT, NAMD2

For manuscripts utilizing custom algorithms or software that are central to the research but not yet described in published literature, software must be made available to editors/reviewers. We strongly encourage code deposition in a community repository (e.g. GitHub). See the Nature Research guidelines for submitting code & software for further information.

Data

Policy information about availability of data

All manuscripts must include a data availability statement. This statement should provide the following information, where applicable:

- Accession codes, unique identifiers, or web links for publicly available datasets
- A list of figures that have associated raw data
- A description of any restrictions on data availability

The published data used in this manuscript are available through following accession codes 5TB4, 5T9R, 5TAP, 5T9V, 5TAL, 5TAQ in Protein Data Bank in Europe (https://www.ebi.ac.uk/pdbe/)
The cryo-EM density maps for states S1 to S6 have been deposited in the Electron Microscopy Data Bank (EMDB) under accession codes EMD-20486, EMD-22393, EMD-22395, EMD-22394, EMD-22396, EMD-22392, and respective model coordinates have been deposited in the Protein Data Bank (PDB) under accession codes 6PV6, 7JMG, 7JMI, 7JMH, 7JMJ, 7JMF. Particle images used in this study have been deposited in the Electron Microscopy Public Image Archive (EMPIAR) under accession code EMPIAR-10315. Cryo-EM density maps and models for all 50 states are available upon request sent to the corresponding authors. Additional supporting information regarding the calculations for the binding affinity of calcium ion in RYR1 using MCCE2 can be found in: https://github.com/SalahBioPhysics/binding_affinity_ryr1.git . Other data are available from the corresponding authors upon reasonable request.

Field-specific reporting

Please select the one below that is the best fit for your research. If you are not sure, read the appropriate sections before making your selection.

[x] Life sciences   [ ] Behavioural & social sciences   [ ] Ecological, evolutionary & environmental sciences

For a reference copy of the document with all sections, see nature.com/documents/nr-reporting-summary-flat.pdf

Life sciences study design

All studies must disclose on these points even when the disclosure is negative.

Sample size	~791,000 single particle Cryo-EM snapshots of RyR1 were used in this study.
Data exclusions	No data were excluded before analysis. By our initial preprocessing (Explained in Method) 20% of the snapshots deemed as outliers and excluded from further steps.
Replication	The biological findings from the outcome of this study were verified by 1) Molecular Dynamics simulations and 2) available RyR1 literature.
Randomization	All data were used to generate energy landscapes. No randomization was needed.
Blinding	There is no group allocation in our study.

Reporting for specific materials, systems and methods

We require information from authors about some types of materials, experimental systems and methods used in many studies. Here, indicate whether each material, system or method listed is relevant to your study. If you are not sure if a list item applies to your research, read the appropriate section before selecting a response.

Materials & experimental systems

n/a	Involved in the study	
[x]	[ ]	Antibodies
[x]	[ ]	Eukaryotic cell lines
[x]	[ ]	Palaeontology
[x]	[ ]	Animals and other organisms
[x]	[ ]	Human research participants
[x]	[ ]	Clinical data

Methods

n/a	Involved in the study	
[x]	[ ]	ChIP-seq
[x]	[ ]	Flow cytometry
[x]	[ ]	MRI-based neuroimaging
